# Indirect improvement of pepino (*Solanum muricatum*) productivity via nitrogen fertilizer-mediated microbial and enzymatic stimulation

**DOI:** 10.3389/fmicb.2025.1612012

**Published:** 2025-08-14

**Authors:** Bingbing Su, Huaidi Pei, Shiweng Li, Zhongming Ma, Yuliang Chen, Yubin Li, Liguang Wang, Minmin Zhang, Zhongwang Li

**Affiliations:** ^1^School of Environmental and Municipal Engineering, Lanzhou Jiaotong University, Lanzhou, China; ^2^Gansu Academy of Agricultural Sciences, Lanzhou, China; ^3^Institute of Biotechnology, Gansu Academy of Agricultural Sciences, Lanzhou, China; ^4^Institute of Forest Fruits and Flowers, Gansu Academy of Agricultural Sciences, Lanzhou, China

**Keywords:** nitrogen fertilizer inputs, soil biochemical properties, nitrogen fertilizer use efficiency, soil microorganism, ginseng fruit (*Solanum muricatum* Aiton)

## Abstract

**Introduction:**

To meet the both escalating production requirements of pepino cultivation and maintaining soil sustainable development through precise exploration of chemical fertilizer input amounts.

**Methods:**

A 5-month greenhouse experiment evaluated how varying nitrogen fertilization rates (0, 75, 150, 225, and 300 kg⋅ha^–1^) modulate soil biochemical properties and their subsequent effects on pepino productivity and fruit nutrients components.

**Results:**

Our study revealed that the N300 treatment maximized vegetative growth (plant height, leaf and fruit dry biomass), as well as plant nitrogen and fruit calcium contents, but significantly reduced root-to-shoot ratio, vitamin C, and soluble sugars versus N0. N225 optimally balanced productivity and nutrition, and elevated nitrogen use efficiency (34.13%), per plant yield (45.60%), fruit protein (142.68%) and calcium (32.72%). N150 showed intermediate benefits with peak stem dry biomass and sugar content, while N75 provided only marginal growth stimulation. Moreover, nitrogen fertilization differentially modified soil biochemical properties, N300 treatment markedly enhanced urease (143.24%), nitrate reductase (99.38%), and sucrase (23.87%) activities, while increasing the relative abundances of *Nitrosomonas* and *Ensifer*, though at the cost of reduced pH, nitrite reductase, and alkaline phosphatase activities. N225 treatment improved microbial ACE and Chao indexes, and enriched the *Opitutus*, but depleted available nitrogen (−29.53%) and available potassium (−27.90%). N150 boosted the relative abundance of *Bacillus* (45.15%), *Arthrobacter* (72.67%), *Sphingomonas* (57.55%), and enriched the *Mesorhizobium*. N75 had slightly positive effects on core genera and nitrogen cycling microorganism. Therefore, we recommend nitrogen application rates of 150 ∼ 225 kg⋅ha^–1^ to optimize pepino production. Moreover, the PLS analysis illustrated that nitrogen fertilization indirectly enhanced pepino productivity by stimulating urease and nitrate reductase activities, and enriching functional microbiota (*Nitrosomonas*, *Opitutus*, *Ensifer*, and *Mesorhizobium*) to facilitate soil nutrient mobilization (soil total nitrogen, available nitrogen and available potassium) for plant growth. Notably, fruit nutrients components (protein and calcium contents) were directly modulated by nitrogen application amounts.

**Discussion:**

Our research provided crucial theoretical foundations for both sustainable soil management and meeting the escalating production requirements in pepino cultivation.

## 1 Introduction

The intensive use of agricultural practices has led to increasing reliance on chemical fertilizers to sustain crop yields in the face of growing global food demands (Sabir et al., 2021; Vries et al., 2022). However, the excessive and repeated application of chemical fertilizers, coupled with low nitrogen use efficiency by crops, has raised significant environmental concerns. For instance, inappropriate fertilizer practices might contribute to soil acidification, promote soil degradation, and lead to the accumulation of excess nutrients (such as phosphorus element) in soil (Ouyang et al., 2020; Sabir et al., 2021; Vries et al., 2022). Furthermore, previous researches indicated that widespread chemical fertilizer application disrupted the diversity, composition, and metabolic functions of soil microbial communities, which are negatively correlated with both crop productivity and quality (Sabir et al., 2021; Park et al., 2023; Zhou et al., 2023).

Fertilization is widely employed to improve soil fertility and enhance crop yields (Ai et al., 2012; Guo et al., 2020). Additionally, fertilizer application increased soil nutrient availability, which promoted the microbial growth (Geisseler and Scow, 2014). However, fertilizer inputs inevitably altered soil properties (Li et al., 2019), subsequently influenced the structure of soil bacterial and fungal communities (Leff et al., 2015; Guo et al., 2020). For instance, nitrogen fertilization, directly affected soil bacterial richness, but indirectly modified bacterial community composition through soil acidification (Zeng et al., 2016). Microbial diversity and community structure undergo significant shifts with the soil nutrient levels rising (Eo and Park, 2016), often favoring fast-growing copiotrophic bacteria at the expense of slower-growing oligotrophs, which typically dominate nutrient-limited soils (Fierer et al., 2011; Ramirez et al., 2012). Furthermore, soil enzymes, produced by microorganisms as metabolic byproducts, play a vital role in organic matter decomposition and nutrient cycling (Islam et al., 2010; Wu et al., 2024), their activities are essential for maintaining soil fertility and overall ecosystem functioning (Manuel Delgado-Baquerizo et al., 2016). Therefore, understanding the effects of fertilization on keystone microbial genera was crucial for developing sustainable fertilization strategies (Wu et al., 2023).

The rhizosphere, a critical soil zone surrounding plant roots, plays a central role in plant nutrition, health, and agricultural productivity (Berg and Smalla, 2009). This dynamic “hotspot” exhibits heightened microbial activity, greater microbial abundance, and intensified interactions among plants, microbes, and soil (Bulgarelli et al., 2013). Rhizosphere microorganisms are widely regarded as fundamental drivers of sustainable agricultural development (Yadav et al., 2018), and also are essential for crop productivity and soil health, as they can regulate organic matter decomposition and enhance nutrients mineralization rates (Barrios, 2007; Yang et al., 2022). Moreover, plant species could influence rhizosphere microbial composition, and foster distinct microbial populations (Berg and Smalla, 2009). Through the secretion of diverse metabolites, plants actively shape the functional dynamics of rhizosphere microbial communities, which in turn feedback on plant growth and productivity (Berendsen et al., 2012; She et al., 2018; Wang et al., 2018).

The pepino (*Solanum muricatum*) is a diploid (2n = 24) perennial herbaceous species within the *Solanaceae* family, closely relates to economically important crops such as tomato (*Solanum lycopersicum*) and potato (*Solanum tuberosum*) (Contreras et al., 2016; Contreras et al., 2017; Yue et al., 2020). Native species is indigenous to South America, with its center of origin in the Andean regions of Peru and Chile, and exhibits a broad geographical distribution ranging from Colombia to Bolivia (Contreras et al., 2016; Contreras et al., 2017). At optimal ripeness, pepino fruit demonstrates considerable nutritional value, containing significant levels of essential minerals (calcium, phosphorus, and potassium) as well as ascorbic acid (Vitamin C), making them a nutritionally valuable dietary component (Zhao et al., 2021; Hou et al., 2025). Furthermore, pepino has gained recognition for its potential health benefits, including demonstrated anti-inflammatory, anticarcinogenic, and antidiabetic properties, which contribute to its growing reputation as a functional food (Prohens et al., 2005; Sudha et al., 2012; Sun et al., 2022).

From 1988 year to the present, some research articles have mainly focused on the developing or postharvest fruit, such as the changes of fruit physiology (Heyes et al., 1994; Martínez-Romero, 2003), nutritional composition (Huyskens-Keil et al., 2006; Herraiz et al., 2016), and metabolic processes (Schaffer et al., 1989; Yue et al., 2020; Yang et al., 2023). Additionally, some studies have evaluated suitable cultivation regions (Hou et al., 2023), pest and disease identification in pepino (Kim et al., 2017; Ishikawa and Takahata, 2019; She et al., 2021), as well as the effects of water and salt stress on growth and development (Chen et al., 1999; Pacheco et al., 2021). Moreover, previous studies have established the phenological stages of open-field cultivated pepino on the Qinghai-Tibet Plateau, providing valuable references for research and cultivation (Hou et al., 2025).

In recent years, pepino has emerged as a valuable crop for agricultural restructuring and farmer income enhancement. Its cultivation has been recently introduced to the Hexi corridor of Gansu Province, where it is primarily grown in controlled greenhouse environments featuring single-stem pruning systems and high-density planting arrangements within limited cultivation areas, while the influence of chemical fertilizer on pepino rhizosphere soil biochemical properties, plant growth and fruit quality remain poorly understood. To address this knowledge gap, we investigated the effects of varying nitrogen fertilizer rates on pepino cultivation, with the following objectives: (1) to determine the optimal nitrogen application rate for pepino production, and (2) to elucidate the microbial mechanism of nitrogen fertilization inputs regulate pepino growth and fruit nutrients components. Our research will provide crucial theoretical foundations for both sustainable soil management and meeting the escalating production requirements of pepino cultivation.

## 2 Materials and methods

### 2.1 Experimental design

The test soil was collected from the experimental crop field of Gansu Provincial Academy of Agricultural Sciences, characterized as loessal soil (a typical aeolian deposit in arid and semi-arid regions) with a bulk density of 1.12 g/cm^3^, the soil mass of per mu is 150,000 kg with a 20 cm depth of topsoil. Test soil samples were obtained prior to fertilizer application for the analysis of fundamental physicochemical properties. The initial soil analysis revealed the following characteristics: organic matter content of 13.57 g⋅kg^–1^, available nitrogen content of 80.87 mg⋅kg^–1^, available potassium content of 137.00 mg⋅kg^–1^, available phosphorus content of 9.44 mg⋅kg^–1^, total nitrogen content of 0.72 g⋅kg^–1^, total phosphorus content of 0.77 g⋅kg^–1^, total potassium content of 19.50 g⋅kg^–1^, and soil pH value of 8.08.

The experimental material consisted of uniformly developed pepino (*Solanum muricatum*) plantlets derived from virus-free tissue-cultured seedlings. The study was conducted in a controlled greenhouse environment at the Institute of Biotechnology, Gansu Provincial Academy of Agricultural Sciences, during the growth period from August 1 to December 31, 2023 (mean temperature: 23.3 ± 0.5°C; mean relative humidity: 37.9 ± 2.1%). Selected seedlings of uniform size were transplanted into ecological pots (30 cm height × 25 cm inner diameter) containing 10 kg of air-dried, sieved loess soil, with one plant per pot.

Five nitrogen application rates were established: 0 (N0), 75 (N75), 150 (N150), 225 (N225), and 300 kg⋅ha^–1^ (N300). According to the formulas 1 ha = 15 mu, 1 mu = 15 × 10^4^ kg soil, the unit conversions of nitrogen fertilizer application rates as Supplementary Table 1. Each treatment consisted of thirty replicate pots, and with three biological replicates allocated for parameter measurements after 150 days. Fertilizers used included urea (≥ 46% TN), granular single superphosphate (≥ 12% P_2_O_5_), and potassium sulfate (≥ 50% K_2_O) for agricultural use. Nitrogen application was split into two stages: 40% during main stem elongation and 60% at fruit set, and delivered via fertigation. Phosphorus (180 kg P_2_O_5_/ha) and potassium (187.5 kg K_2_O/ha) fertilizers were uniformly incorporated into the soil prior to potting as cultivation soil.

### 2.2 Plant sample collection and measurement at pepino maturity stage

Plant height was measured from the stem base to the apical meristem using a ruler. Stem diameter was determined at 2 cm above the stem base using digital calipers. Pepino plants were carefully separated into root, stem, leaf, and fruit components for biomass analysis. The samples were first heat-treated at 105°C for 30 min to terminate enzymatic activity, then dried at 80°C in a forced-air oven until constant weight was achieved. The dry weight of stem, leaf, and fruit components were measured using digital scale. Plant total nitrogen content was determined using the Kjeldahl method (Kjeltec™ 8200 Semi-Automatic Nitrogen Analyzer, FOSS, Sweden).

The total N uptake was calculated as the sum of N uptake by all the organs of the plant. To evaluate fertilization efficiency, the nitrogen fertilizer use efficiency (NUE) was calculated as follows (Ma et al., 2024):


Total⁢N⁢uptake⁢(g/plant)=

Total⁢N⁢content⁢(g/kg)×Dry⁢matter⁢weight⁢(kg/plant)

NUE(%)=

$$\frac{\mathrm{Total}\,N\,\mathrm{uptake}-\mathrm{Total}\,N\,\mathrm{uptake}\,\mathrm{in}\,\mathrm{N0}\,\mathrm{treatment}}{\mathrm{Applied}\,N}\times 100$$

According to the standard test methods of the Chinese national food standard, the protein content of pepino was determined by Kjeldahl method (Kjeltec™ 8200 Semi-Automatic Nitrogen Analyzer, FOSS, Sweden). The vitamin C content was determined by fluorescence method (Shimadzu RF-540 Spectrofluorophotometer, Shimadzu Corporation, Japan). The crude fiber content was determined by acid-alkali washing method. The soluble sugar content was determined by direct titration. The calcium element was determined by inductively coupled plasma mass spectrometry (Thermo Scientific™ iCE 3500 Atomic Absorption Spectrometer, Thermo Fisher Scientific, United States). The selenium element was determined by hydride atomic fluorescence spectrometry (RGF-8740 Atomic Fluorescence Spectrophotometer, Beijing Rayleigh Analytical Instruments Corp., Beijing, China).

### 2.3 Soil sample collection and measurement at pepino maturity stage

For each pepino plant, the complete root system was carefully extracted from its pot. Rhizosphere soil was collected by gently shaking the roots to dislodge soil particles adhering to the root surface, followed by removal of visible impurities (e.g., plant debris) (Ren et al., 2022). The rhizosphere soil samples were collected and mixed in sterile self-sealing bags, and then divided into two parts, one part sample was stored in 5 mL cryovials at −*80*°C until DNA extraction; the other portion of soil sample had been naturally dried and sieved, was used for the determination of soil chemical properties and enzymatic activity (based on the measurement of dry soil method) over 1 month.

According to the standard test methods of the Chinese national standard, soil organic matter (SOM) content was determined by potassium dichromate oxidation method (Walkley, 1935). The available nitrogen (AN) was determined by the alkaline diffusion method. Available phosphorus (AP) was extracted by sodium bicarbonate and quantified with a spectrophotometer (Varian Cary 50 UV-Vis Spectrophotometer, Agilent Technologies, United States). Available potassium (AK) was extracted by ammonium acetate and quantified by a flame photometer (Sherwood Model 410 Flame Photometer, Sherwood Scientific Ltd., United Kingdom). The total nitrogen (TN) was determined by Kjeldahl method (Kjeltec™ 8200 Semi-Automatic Nitrogen Analyzer, FOSS, Sweden). Total phosphorus (TP) content was determined by alkali fusion-molybdenum antimony colorimetric method (Varian Cary 50 UV-visible spectrophotometer, Agilent Technologies, United States). The total potassium (TK) content was determined by alkali fusion-flame photometry (Sherwood M410 Flame Photometer, Sherwood Scientific Ltd., United Kingdom). The pH measurement was performed after oscillation for 30 min, and the ratio of soil to water (W/V) was 1:2.5 (pHS-25 pH Meter, Shanghai Precision and Scientific Instrument Co., Ltd., Shanghai, China) (Li et al., 2023).

Soil urease activity was measured by sodium phenol-sodium hypochlorite colorimetric method (Xu et al., 2021). Soil sucrase activity was measured by 3, and 5-dinitro salicylic acid colorimetry, and its enzyme activity was expressed as the milligrams of glucose produced in 1 g of soil over a 24 h period (Gao et al., 2013). Soil nitrate reductase activity was determined by the phenol disulfonic acid colorimetric method (Hu et al., 2014). Soil nitrite reductase activity was determined by an enzymatic assay. Soil alkaline phosphatase activity was determined by the colorimetric method using disodium benzene phosphate.

### 2.4 DNA extraction, amplicon sequencing, and bioinformatics analysis

Total microbial genomic DNA was extracted from pepino rhizosphere soil samples using the FastPure Soil DNA Isolation Kit (Magnetic bead) (MJYH, Shanghai, China) following the manufacturer’s protocol. DNA quality and concentration were assessed through 1.0% agarose gel electrophoresis and a NanoDrop2000 spectrophotometer (Thermo Scientific, United States), with subsequent storage at −80°C until further analysis. The hypervariable V3-V4 region of the bacterial 16S rRNA gene was amplified using primer pairs 338F (5′-ACTCCTACGGGAGGCAGCAG-3′) and 806R (5′-GGACTACHVGGGTWTCTAAT-3′) (Liu et al., 2015) in a T100 Thermal Cycler PCR system (Bio-Rad, United States).

The PCR reaction mixture including 4 μL of 5 × Fast Pfu buffer, 2 μL of 2.5 mM dNTPs, 0.8 μL of each primer (5 μM), 0.4 μL of Fast Pfu polymerase, 10 ng of template DNA, and ddH_2_O to a final volume of 20 μL. Amplification was performed under the following conditions: initial denaturation at 95°C for 3 min; 27 cycles of denaturation at 95°C for 30 s, annealing at 55°C for 30 s, and extension at 72°C for 45 s; followed by a final extension at 72°C for 10 min and cooling to 4°C. PCR products were separated by 2% agarose gel electrophoresis, purified using the PCR Clean-Up Kit (YuHua, Shanghai, China) according to the manufacturer’s instructions, and quantified using a Qubit 4.0 fluorometer (Thermo Fisher Scientific, United States). Equimolar concentrations of purified amplicons were pooled and subjected to paired-end sequencing on an Illumina NextSeq 2000 platform (San Diego, United States) by Majorbio Bio-Pharm Technology Co., Ltd. (Shanghai, China), following standard protocols.

The raw FASTQ files were demultiplexed using an in-house Perl script, followed by quality filtering with fastp (version 0.19.6) (Chen et al., 2018) and merging using FLASH (version 1.2.7) (Magoč and Salzberg, 2011) under the following parameters: (i) The reads were truncated at any site receiving an average quality score of < 20 over a 50 bp sliding window, and the truncated reads shorter than 50 bp were discarded, reads containing ambiguous characters were also discarded; (ii) Only overlapping sequences longer than 10 bp were assembled according to their overlapped sequence. The maximum mismatch ratio of overlap region is 0.2, reads that could not be assembled were discarded; (iii) Samples were distinguished according to the barcode and primers, and the sequence direction was adjusted, exact barcode matching, 2 nucleotide mismatches in primer matching. Then the optimized sequences were clustered into operational taxonomic units (OTUs) using UPARSE (version 7.1) with 97% sequence similarity level (Stackebrandt and Goebel, 1994; Edgar, 2013). The taxonomy of each OTU representative sequence was analyzed by RDP Classifier version 2.2 (Wang et al., 2007) against the 16S rRNA gene database (SILVA_v138) using confidence threshold of 0.7.

### 2.5 Statistical analysis

The column plots and tables were generated using Excel 2016 and Origin Pro 2021. SPSS 26.0 statistical software was used, and Duncan method of one-way (ANOVA) analysis was used to detect significant differences (*P* < 0.05). Soil bioinformatics analysis was performed using the Majorbio Cloud platform (Majorbio, Shanghai, China). Alpha diversity indices (ACE and Chao1 richness indices (Chao, 1984), Shannon (Shannon, 1948) and Simpson (Simpson, 1949) diversity indices) were calculated from OTU data using Mothur v1.30.1 (Schloss et al., 2009). Beta diversity was assessed through principal coordinate analysis (PCoA) based on Bray-Curtis dissimilarity metrics. The correlation heatmap was generated based on Pearson correlation analysis. Mantel tests examining soil microbial community structure-biochemical property relationships were implemented using the “ggcor” package in R (version 4.4.1) (Du et al., 2023). Factor importance analysis was conducted with the “rfPermute” package in R (Chen et al., 2021). Differential species analysis employed LEfSe (Linear Discriminant Analysis Effect Size) with an LDA threshold > 2.5. Partial Least Squares Path Modeling (PLS-PM) was performed using the “plspm” R package.

## 3 Results analysis

### 3.1 Effect of nitrogen fertilizer rates on pepino growth at maturity stage

#### 3.1.1 Nitrogen fertilizer addition boosted the pepino growth parameters

Effect of nitrogen fertilizer inputs on pepino growth parameters at maturity stage (Table 1), which demonstrated that increasing nitrogen fertilizer inputs progressively enhanced the pepino plant height, leaf dry biomass, fruit dry biomass. Compared to N0 control, N150, N225, and N300 treatments significantly increased plant height by 16.20, 26.00, and 26.36 cm, leaf dry biomass by 68.84, 101.40, and 113.79%, and fruit dry biomass by 29.10, 45.54, and 48.21%, respectively.

**TABLE 1:** Effect of nitrogen fertilizer rates on pepino growth parameters at maturity stage.

Treatments	Plant height (cm)	Stem diameter (mm)	Leaf dry biomass (g)	Stem dry biomass (g)	Root dry biomass (g)	Fruit dry biomass (g)	Root to shoot ratio (R/T)
N0	57.67 c ± 8.62	7.61 a ± 0.93	7.83 c ± 1.27	6.07 c ± 1.34	3.26 b ± 0.43	22.40 b ± 2.04	0.089 a ± 0.004
N75	64.67 bc ± 4.28	7.86 a ± 0.65	9.46 c ± 0.45	7.04 bc ± 0.45	3.61 ab ± 0.60	26.50 ab ± 4.78	0.086 a ± 0.015
N150	73.87 ab ± 3.42	8.12 a ± 0.41	13.22 b ± 1.76	9.93 a ± 0.71	4.60 a ± 0.28	29.10 a ± 3.96	0.093 a ± 0.005
N225	83.67 a ± 9.50	8.43 a ± 1.17	15.77 ab ± 0.35	9.67 a ± 0.88	3.90 ab ± 0.42	32.60 a ± 1.72	0.062 b ± 0.011
N300	84.03 a ± 5.82	8.56 a ± 0.48	16.74 a ± 2.46	8.67 ab ± 1.10	3.70 ab ± 0.76	33.20 a ± 3.93	0.063 b ± 0.012

Different lowercase letters indicate the significant differences between nitrogen fertilizer rates and pepino cultivation (*P* < 0.05). Tables are the mean ± standard error (*n* = 3). N0, N75, N150, N225 and N300 represent chemical nitrogen fertilizer levels, i.e., 0 kg⋅ha^–1^, 75 kg⋅ha^–1^, 150 kg⋅ha^–1^, 225 kg⋅ha^–1^, and 300 kg⋅ha^–1^, respectively. The same below.

Stem dry biomass and root dry biomass exhibited the increasing and then decreasing with increasing of nitrogen levels, peaking at 9.93 g and 4.60 g in N150 treatment, respectively. Moreover, the root-to-shoot ratio significantly decreased by 30.34 and 29.21% in N225 and N300 treatments. The stem diameter showed minimal variation across treatments.

#### 3.1.2 Nitrogen fertilizer addition improved the pepino productivity

**FIGURE 1:**
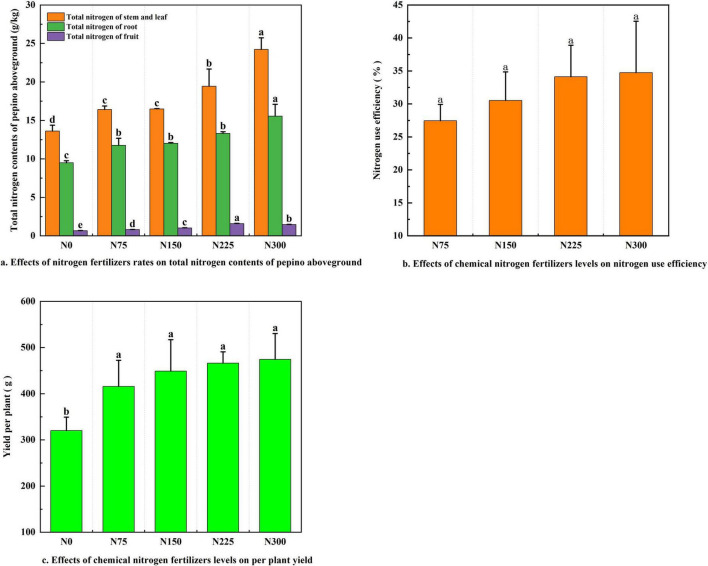
Effect of nitrogen fertilizer addition on pepino nitrogen use efficiency and yield at maturity stage. **(a)** Effects of nitrogen fertilizers rates on total nitrogen contents of pepino aboveground. **(b)** Effects of nitrogen fertilizers levels on fertilizers use efficiency. **(c)** Effects of chemical nitrogen fertilizers levels on per plant yield.

Nitrogen fertilizer inputs significantly increased total nitrogen content in pepino stems and leaves, roots, and fruits, compared to the N0 control (Figure 1a). The N300 treatment resulted in the highest nitrogen content in stems and leaves (24.23 g⋅kg^–1^) and roots (15.55 g⋅kg^–1^). In contrast, N225 treatment led to the highest fruit nitrogen content (1.59 g⋅kg^–1^).

Nitrogen fertilizer application significantly enhanced both the nitrogen use efficiency (NUE) and yield per plant compared to the N0 control (Figures 1b,c, *P* < 0.05). The N75, N150, N225, and N300 treatments increased NUE by 27.46, 30.52, 34.13, and 34.74%, respectively, while yield per plant rose by 29.90, 40.21, 45.60, and 48.16%. Although the N300 treatment showed the highest values of NUE and yield, the improvement over N225 was negligible. Thus, from the perspective of cost-saving and efficiency improvement, N225 treatment achieved optimal performance with an NUE of 34.13% and a per plant yield of 466.02 g.

#### 3.1.3 Nitrogen fertilizer addition enhanced the pepino nutrient components

Appropriate nitrogen fertilizer amounts significantly improved several key quality parameters of pepino fruit (Table 2). Vitamin C content increased significantly by 13.59, 27.18, and 41.79% under the N75, N150, and N225 treatments, respectively, but the N300 treatment resulted in a 16.67% reduction compared to N0 control. Soluble sugar content followed a quadratic response to nitrogen application, peaking at 8.25 g⋅100g^–1^ under N150 treatment, with a 7.84% increase than N0. Calcium content exhibited a linear increase with nitrogen application, with the N300 treatment showing the highest accumulation (100.35% greater than N0). Conversely, nitrogen fertilizer inputs had minimal effects on crude fiber and selenium contents, which remained a relatively stable value across all treatments.

**TABLE 2:** Effect of nitrogen fertilizer inputs on pepino quality parameters at maturity stage.

Treatments	Vitamin C (mg⋅100 g^–1^)	Protein (g⋅100 g^–1^)	Soluble sugar (g⋅100 g^–1^)	Coarse fiber (%)	Calcium (mg⋅kg^–1^)	Selenium (mg⋅kg^–1^)
N0	19.50 d ± 1.20	0.410 e ± 0.020	7.65 b ± 0.25	0.65 a ± 0.05	57.15 c ± 6.65	0.0067 a
N75	22.15 c ± 0.25	0.515 d ± 0.005	8.15 a ± 0.15	0.75 a ± 0.05	73.45 b ± 4.45	0.0108 a
N150	24.80 b ± 2.10	0.635 c ± 0.015	8.25 a ± 0.15	0.55 a ± 0.05	73.9 b ± 8.90	0.0070 a
N225	27.65 a ± 1.05	0.995 a ± 0.025	7.70 ab ± 0.01	0.60 a ± 0.10	75.85 b ± 2.85	0.0067 a
N300	16.25 e ± 1.85	0.920 b ± 0.020	7.35 b ± 0.25	0.70 a ± 0.10	114.5 a ± 7.5	0.0084 a

### 3.2 Effect of nitrogen fertilizer rates on soil chemical properties at maturity stage

#### 3.2.1 Soil nutrient contents

Table 3 demonstrated that nitrogen fertilizer addition differentially affects various soil chemical parameters, with most pronounced changes occurring at application rates of 150 ∼ 300 kg⋅ha^–1^. Compared to the N0 control, the application of nitrogen fertilizer decreased soil pH (*p* < 0.05 for N300 treatment), available nitrogen (AN, *p* < 0.05 for N150 ∼ N300 treatments), available potassium (AK, *p* < 0.05 for all treatments), and total nitrogen (TN, *p* < 0.05 for N150 ∼ N300 treatments). Conversely, a significant increase in available phosphorus (AP) content. As for soil organic matter (SOM), N75 and N150 treatments slightly decreased SOM content, but N225 and N300 treatments slightly improved SOM content. There no clearly impact on total phosphorus (TP) and total potassium (TK), except for a significant increase under N300 treatment.

**TABLE 3:** Effect of chemical nitrogen fertilizer amounts on soil chemical properties.

Treatments	pH	SOM(g/kg)	AN(mg/kg)	AP(mg/kg)	AK(mg/kg)	TN(g/kg)	TP(g/kg)	TK(g/kg)
N0	7.95 a ± 0.08	12.70 ab ± 0.10	82.97 a ± 5.45	11.50 c ± 0.50	128.33 a ± 6.03	0.71 a ± 0.01	0.99 a ± 0.02	20.60 b ± 0.75
N75	7.89 ab ± 0.03	12.00 b ± 0.69	78.67 a ± 7.36	17.25 a ± 0.75	108.40 b ± 10.61	0.69 ab ± 0.03	0.99 a ± 0.05	21.47 b ± 0.17
N150	7.85 ab ± 0.02	11.97 b ± 0.15	64.30 b ± 0.70	14.85 b ± 0.75	93.13 c ± 10.33	0.67 b ± 0.03	1.02 a ± 0.04	19.87 b ± 0.21
N225	7.85 ab ± 0.03	14.40 a ± 0.95	58.47 b ± 2.83	14.53 b ± 1.17	92.53 c ± 4.16	0.67 b ± 0.01	1.01 a ± 0.09	20.27 b ± 0.35
N300	7.79 b ± 0.03	13.40 ab ± 2.09	65.93 b ± 2.14	14.63 b ± 1.07	97.80 bc ± 6.02	0.67 b ± 0.02	1.04 a ± 0.01	25.60 a ± 0.44

SOM, is soil organic matter; AN, is available nitrogen; AP, is available phosphorus; AK, is available potassium; TN, is total nitrogen; TP, is total phosphorus; TK, is total potassium; Tables are the mean ± standard error (*n* = 3).

#### 3.2.2 Soil enzyme activities

**FIGURE 2:**
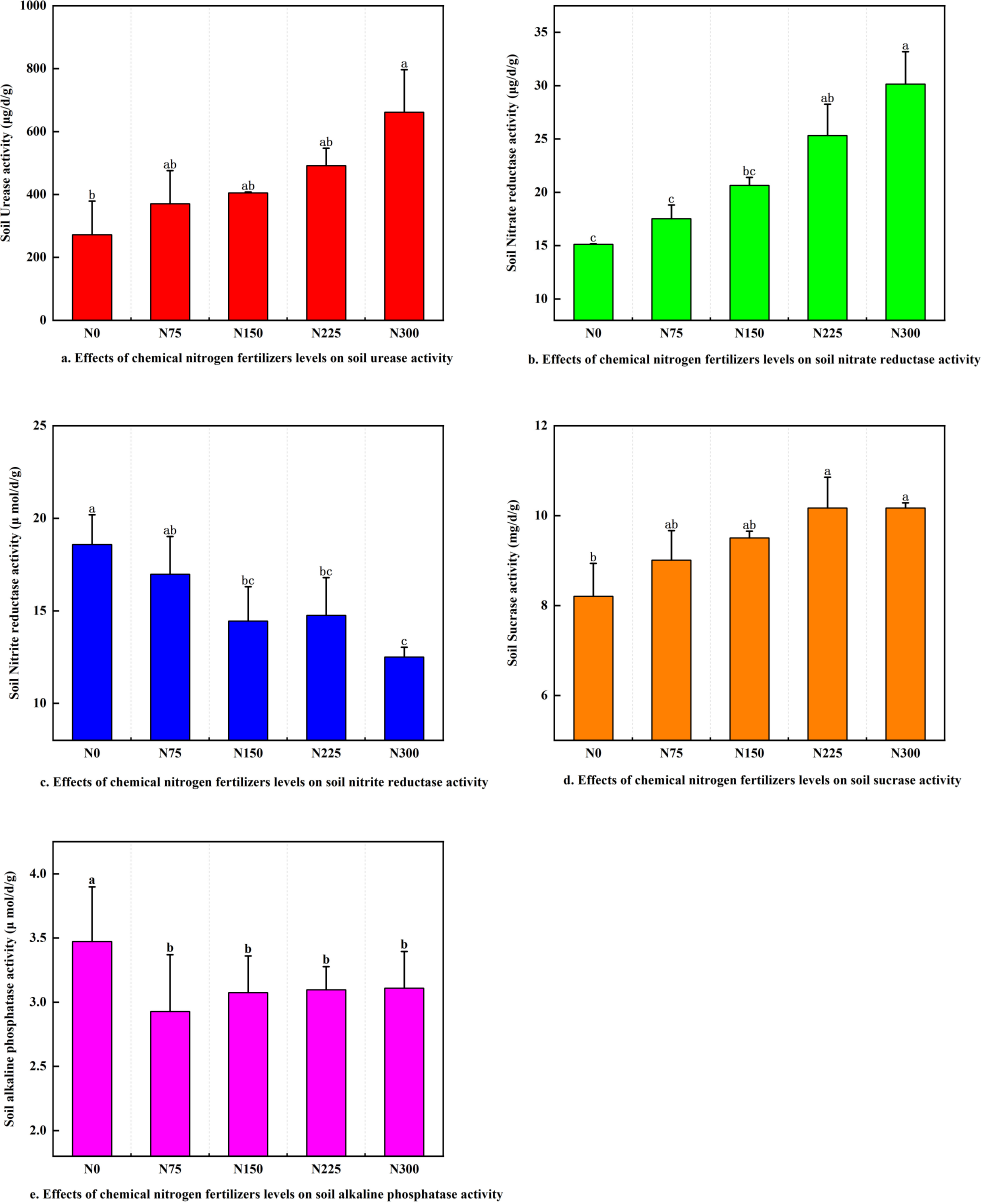
Effect of chemical nitrogen fertilizer levels on soil enzyme activities at maturity stage. **(a)** Effect of chemical nitrogen fertilizers levels on soil urease activity. **(b)** Effect of chemical nitrogen fertilizers levels on soil nitrate reductase activity. **(c)** Effect of chemical nitrogen fertilizers levels on soil nitrite reductase activity. **(d)** Effect of chemical nitrogen fertilizers levels on soil sucrase activity. **(e)** Effect of chemical nitrogen fertilizers levels on soil alkaline phosphatase sucrase activity.

The application of nitrogen fertilizer noticeably enhanced the activities of soil urease, nitrate reductase, and sucrase in a dose-dependent manner (Figures 2a,b,d). Among all treatments, N300 exhibited the highest enzymatic activities, with soil urease, nitrate reductase, and sucrase increasing by 143.24, 99.38, and 23.87%, respectively, compared to the N0 control. In contrast, nitrogen fertilizer inputs markedly suppressed the activities of soil nitrite reductase (Figure 2c), and the N300 treatment recorded the lowest nitrite reductase activity, showing a 26.34% reduction relative to N0. Similarly, nitrogen fertilizer application led to a decline in alkaline phosphatase activity, though no significant differences were observed among the N75 ∼ N300 treatments (Figure 2e).

### 3.3 Effect of nitrogen fertilizer inputs on soil bacterial diversity at maturity stage

#### 3.3.1 Alpha diversity

The ACE and Chao indices of soil bacterial communities initially increased but then slightly decreased with increasing nitrogen fertilizer rates (Table 4). Specifically, the N225 treatment significantly enhanced soil bacterial ACE and Chao indices by 9.27 and 9.88%, respectively, compared to N0, while the N300 treatment showed values similar to those of N0. In contrast, nitrogen fertilizer inputs had no significant effect on the Shannon and Simpson indices, this indicated that chemical nitrogen fertilizer application primarily influenced soil bacterial richness rather than diversity.

**TABLE 4:** Effect of chemical nitrogen fertilizer amounts on soil bacterial alpha diversity at pepino mature stage

Soil microbial	Alpha diversity indices	N0 treatment	N75 treatment	N150 treatment	N225 treatment	N300 treatment
Soil bacteria	ACE	4363.09 b ± 265.06	4433.55 b ± 86.87	4565.61 ab ± 102.82	4767.75 a ± 180.53	4492.31 ab ± 106.45
	Chao	4209.02 b ± 287.94	4306.15 ab ± 80.86	4418.78 ab ± 117.84	4624.84 a ± 158.50	4373.46 ab ± 78.11
	Shannon	6.80 a ± 0.02	6.68 a ± 0.23	6.81 a ± 0.02	6.88 a ± 0.06	6.81 a ± 0.01
	Simpson	0.0032 a ± 0.0001	0.0045 a ± 0.0002	0.0035 a ± 0.0001	0.0030 a ± 0.0002	0.0032 a ± 0.0001

#### 3.3.2 Beta diversity

**FIGURE 3:**
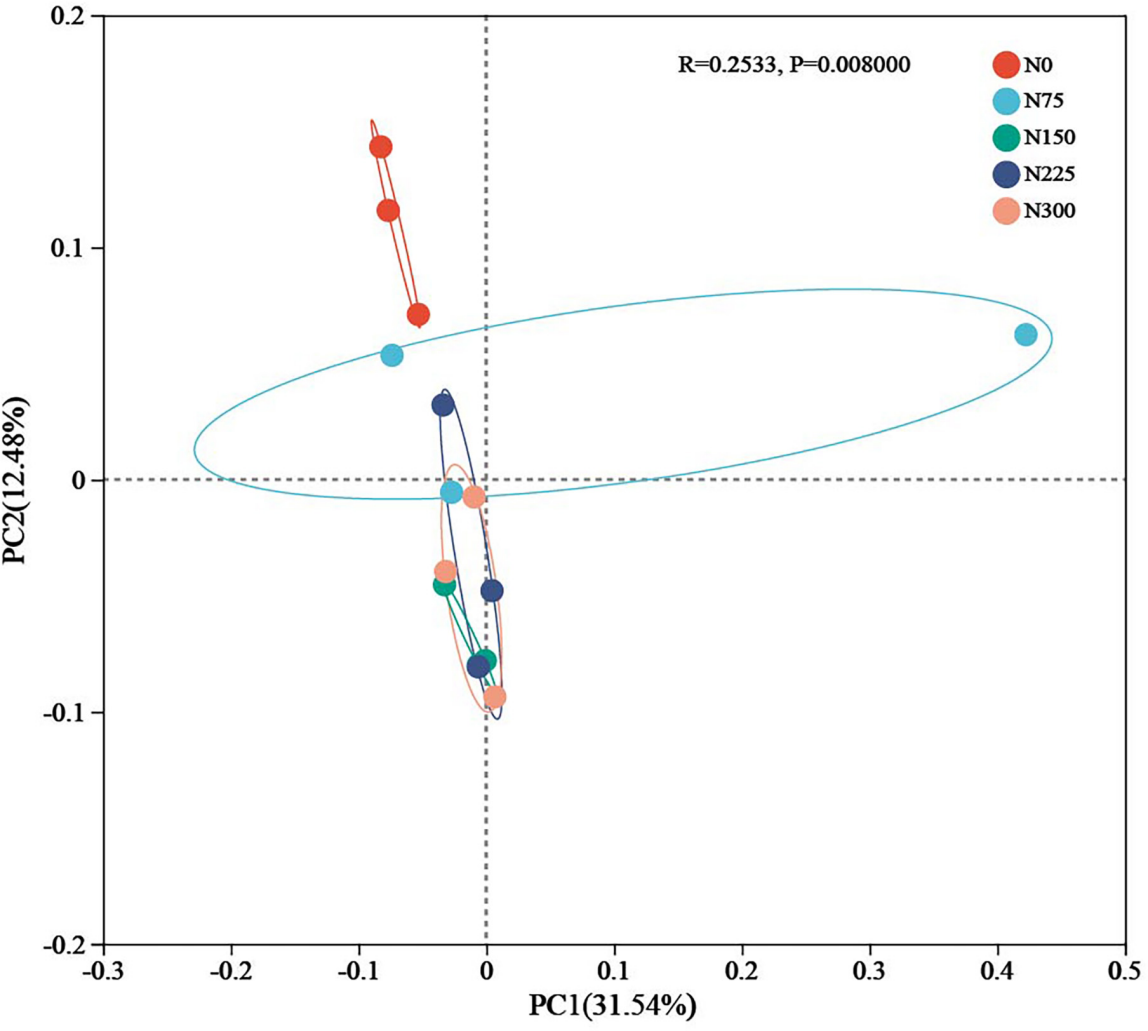
Effect of nitrogen fertilizer rates on soil bacteria beta diversity at maturity period.

Principal coordinates analysis (PCoA) based on Bray-Curtis distance matrix, and coupled with ANOSIM intergroup difference analysis, it was conducted to assess variations in microbial community structure across treatments (Figure 3). Nitrogen fertilizer inputs clearly altered soil bacterial community structure (*P* = 0.008). Notably, the community structures of N150 ∼ N300 treatments clustered closely together but were distinctly separated from the N0 treatment.

### 3.4 Effect of nitrogen fertilizer rates on soil bacterial community composition at maturity stage

**FIGURE 4:**
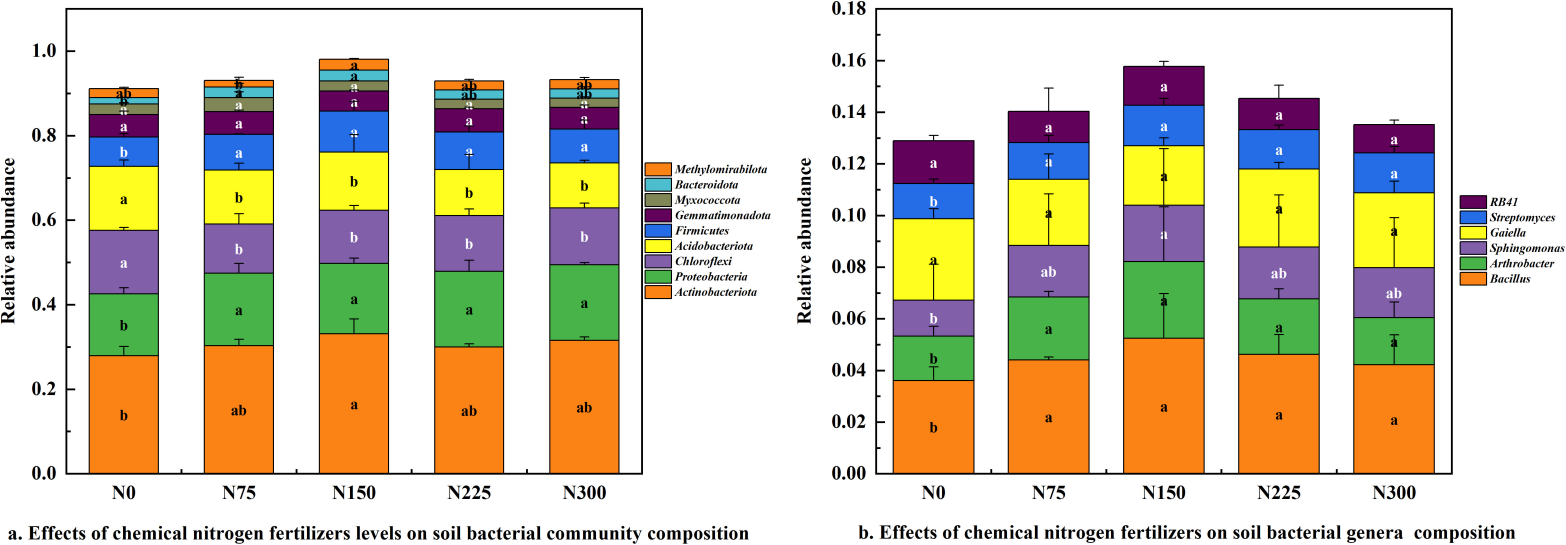
Effect of nitrogen fertilizer rates on soil bacterial community composition at maturity stage. **(a)** Effect of chemical nitrogen fertilizers levels on soil bacterial community composition. **(b)** Effect of chemical nitrogen fertilizers levels on soil bacterial genera composition.

Nitrogen fertilization enhanced the relative abundance of *Actinobacteriota* (7.45 ∼ 18.59%), *Proteobacteria* (13.73 ∼ 22.14%), *Firmicutes* (15.28 ∼ 38.83%), and *Bacteroidota* (46.67 ∼ 75.60%) (Figure 4a). Conversely, nitrogen fertilizer inputs reduced the relative abundance of *Chloroflexi* (−10.24 ∼ −22.43%) and *Acidobacteriota* (−9.24 ∼ −30.09%). Among the treatments, N150 significantly increased the abundance of *Actinobacteriota* (18.59%), *Proteobacteria* (13.73%), *Firmicutes* (38.83%), and *Bacteroidota* (20.47%), compared to N0 treatment.

The dominant bacterial genera in pepino rhizosphere soil included *Bacillus* (3.61 ∼ 5.24%), *Arthrobacter* (1.72 ∼ 2.79%), *Sphingomonas* (1.39 ∼ 2.19%), *Gaiella* (2.30 ∼ 3.15%), *Streptomyces* (1.36 ∼ 1.57%), and *RB41* (1.10 ∼ 1.66%) (Figure 4b). Nitrogen fertilization generally increased the relative abundance of *Bacillus*, *Arthrobacter*, *Sphingomonas*, and *Streptomyces*. Notably, the N150 treatment exhibited the most pronounced stimulatory effect, and significantly enhanced the relative abundance of *Bacillus* (45.15%), *Arthrobacter* (72.67%), *Sphingomonas* (57.55%), and *Streptomyces* (15.44%), compared to N0 control.

### 3.5 The correlations between pepino productivity and fruit quality indices at maturity period

**FIGURE 5:**
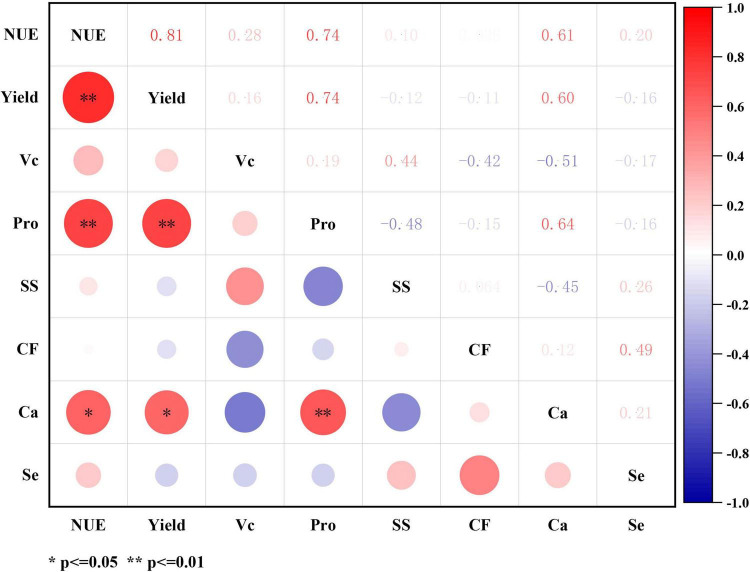
The correlation between pepino productivity and quality indices at maturity stage. Yield is pepino yield of per plant. NUE, is nitrogen use efficiency; Vc, is vitamin C; Pro, is the protein; SS, is soluble sugar; CF, is coarse fiber; Ca, is calcium element; Se, is selenium element. *p ≤ 0.05, **p≤ 0.01.

At pepino maturity period, significant correlations were observed among NUE, yield, and quality indices (Figure 5). Both NUE and yield demonstrated significant positive correlations with calcium (Ca) content, with correlation coefficients of 0.61 and 0.60, respectively. Additionally, both NUE and yield showed highly significant positive correlations with protein (Pro) content (*r* = 0.74). A particularly strong positive correlation was found between NUE and yield (*r* = 0.81). Furthermore, the calcium content in pepino fruits exhibited a highly significant positive correlation with protein content (*r* = 0.64).

### 3.6 The relationships between soil biochemical properties and pepino productivity

**FIGURE 6:**
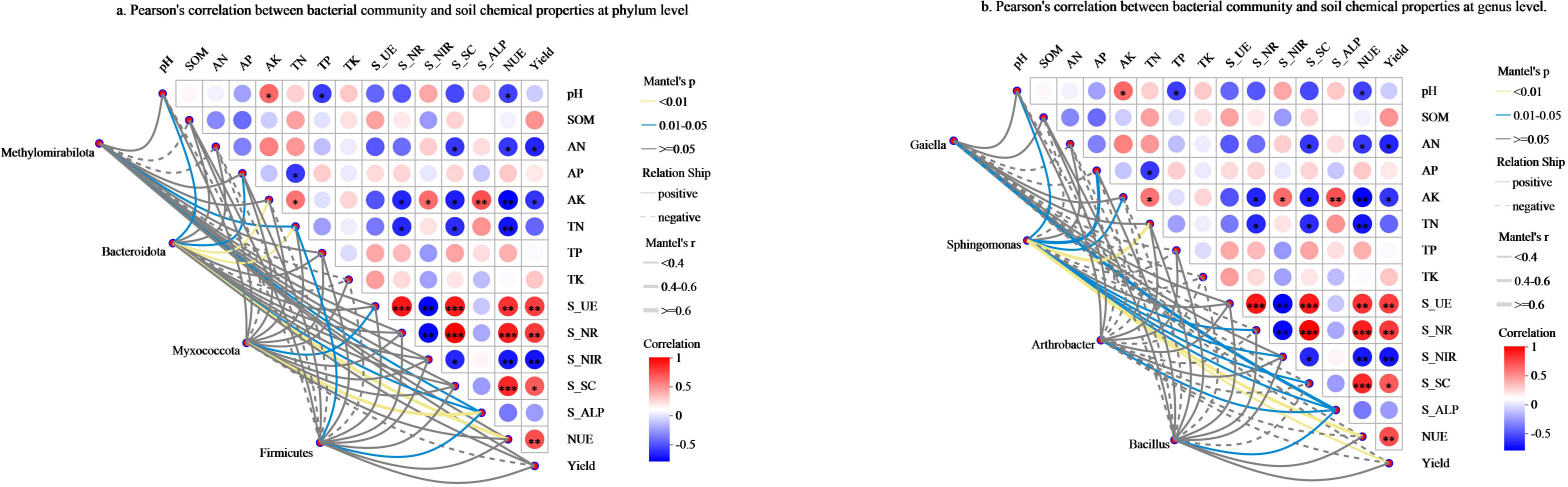
The relationships between soil biochemical properties and pepino productivity. **(a)** Pearson’s correlation between bacterial community and soil chemical properties at phylum level. **(b)** Pearson’s correlation between bacterial community and soil chemical properties at genus level. pH, is soil pH value; SOM, is soil organic matter; AN, is available nitrogen; AP, is available phosphorus; AK, is available potassium; TN, is total nitrogen; TP, is total phosphorus; TK, is total potassium; S_UE, is soil urease; S_NR,is soil nitrate; S_NIR, is soil nitrite; S_SC, is soil sucrase; S_ALP, is soil alkaline phosphatase.

Mantel test analysis was performed to examine the relationships between soil biochemical properties and pepino productivity (Figure 6), which revealed that AK showed a significant positive correlation with S_NIR and S_ALP, while exhibiting a notable negative correlation with S_NR and S_SC. TN was significantly negatively correlated with S_NR and S_SC. AN had a significant negative correlation with S_SC. NUE was notably positively correlated with S_UE, S_NR, and S_SC, but clearly negatively correlated with soil pH, AN, AK, TN, and S_NIR. Yield demonstrated a notably positive correlation with S_UE, S_NR, S_SC, and NUE, while showing a significant negative correlation with AN, AK, and S_NIR.

In addition, *Bacteroidota* showed a significant correlation with pH, AP, and S_ALP (*p* < 0.05), as well as a highly significant correlation with AK, TN, and NUE (*p* < 0.01) (Figure 6a). At the genus level, *Sphingomonas* was significantly correlated with pH, AP, AK, S_NR, S_NIR, S_SC, and S_ALP (*p* < 0.05), while displaying a highly significant correlation with TN, NUE, and yield (*p* < 0.01) (Figure 6b).

### 3.7 The relative importance of soil biochemical properties to NUE and production

**FIGURE 7:**
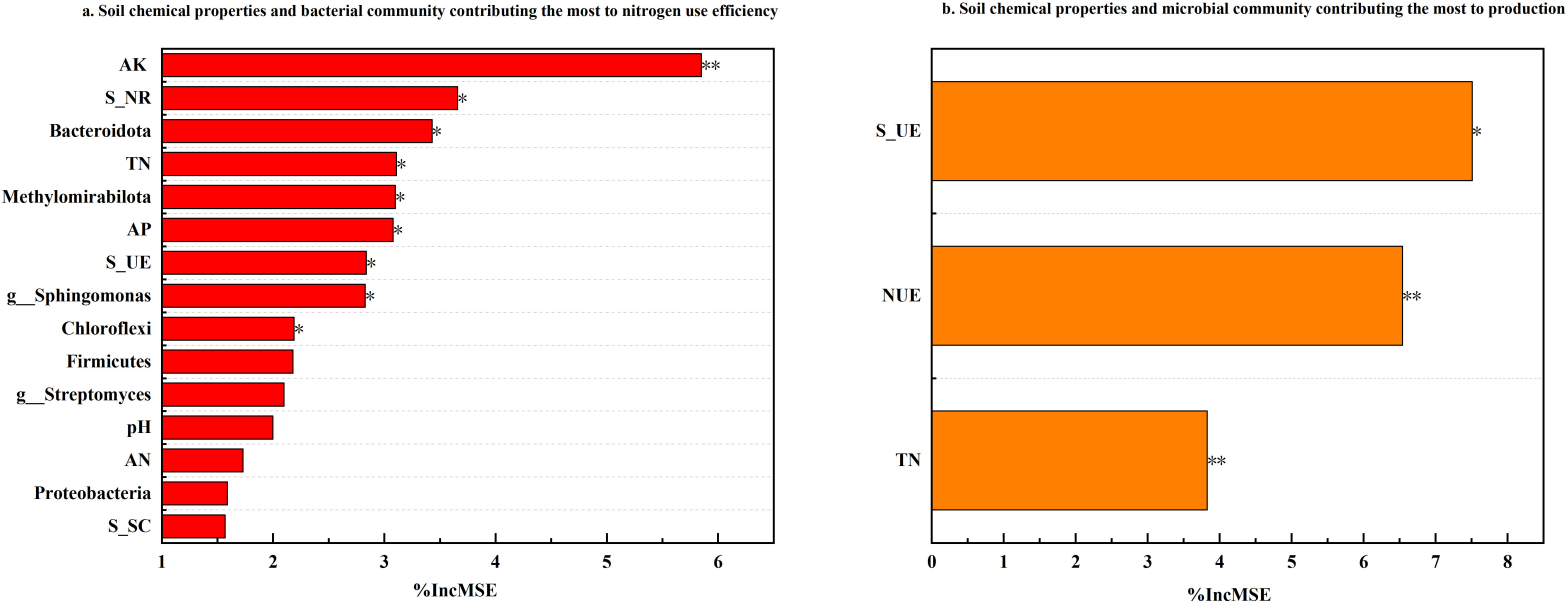
The relative importance of soil biochemical properties to NUE and production. To simplify the figure, the top fifteen variables contributing the most to nitrogen use efficiency accrual are shown according to the result of the random forest analysis, and the top three variables contributing the most to production accrual are shown according to the result of the random forest analysis. The significant statistical results were labeled (*P < 0.05, **P < 0.01).

The random forest algorithm was employed to assess and rank the relative importance of various factors influencing pepino nitrogen use efficiency (NUE) and production (Figure 7). Analysis revealed that soil microbial communities and chemical properties played predominant roles in determining NUE, among all evaluated parameters, AK emerged as the most significant factor affecting NUE (%IncMSE = 5.85), followed sequentially by S_NR, TN, AP, S_UE, and the relative abundance of *Bacteroidota*, *Methylomirabilota*, *Sphingomonas*, and *Chloroflexi*. As for production, S_UE demonstrated the highest importance (%IncMSE = 7.51), with NUE (%IncMSE = 6.54) and TN (%IncMSE = 3.83) being the subsequent most influential factors.

### 3.8 Functional microorganism analysis

The LEfSe (LDA score > 2.5) analysis revealed distinct microbial taxa across nitrogen addition treatments (Supplementary Figure 1). The N225 treatment was characterized by unique taxa such as *Nitrosospira*, while N300 was associated with taxa including *Nitrosomonas*, *Ensifer*, and *Rhizobiaceae*.

**FIGURE 8:**
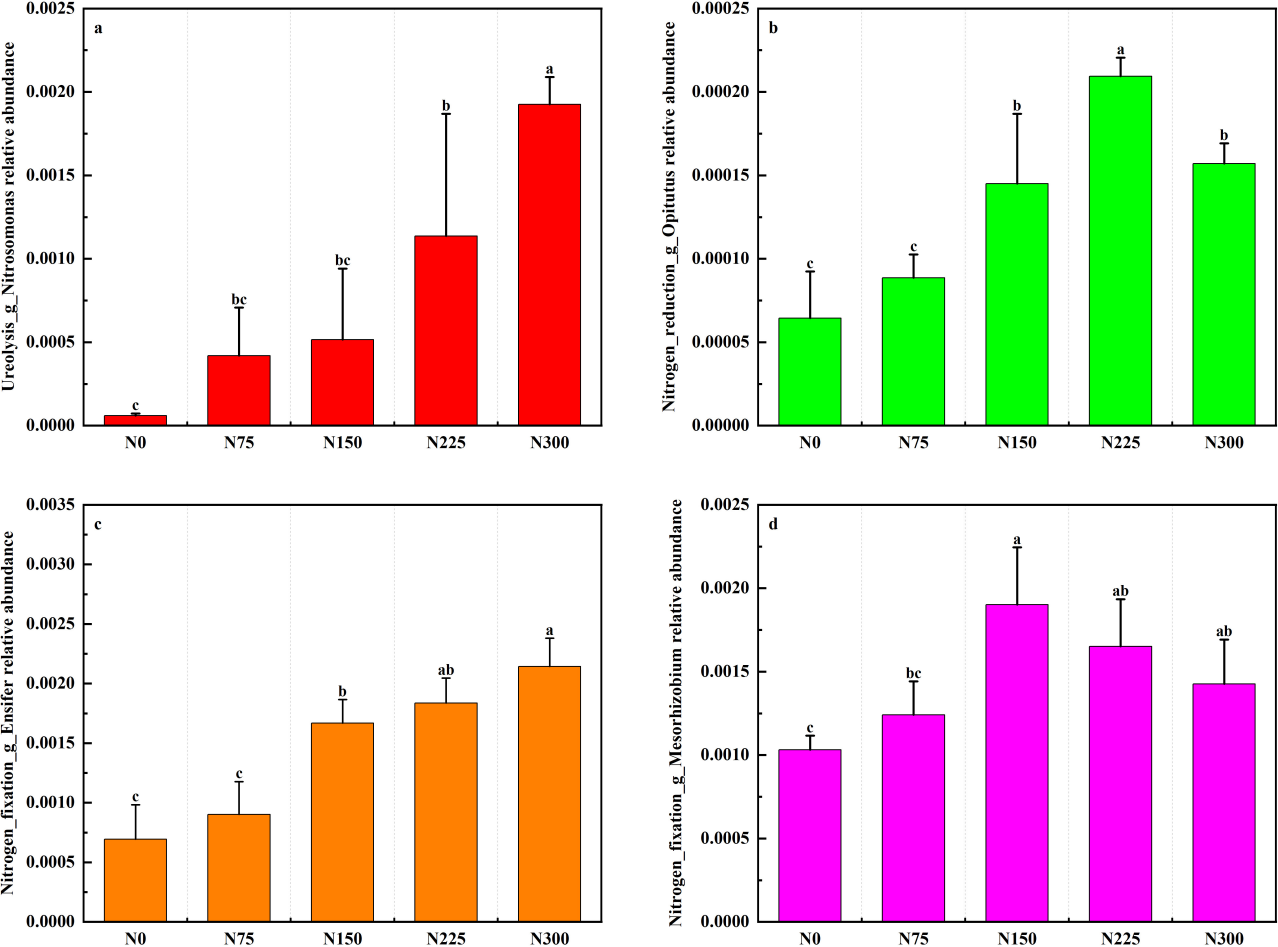
Effect of nitrogen fertilizer inputs on the relative abundance of functional microorganisms at maturity period.

Nitrogen fertilization enhanced the relative abundance of functional microorganisms involved in nitrogen cycling (Figure 8). Specifically, N300 treatment showed the highest relative abundance of *Nitrosomonas* and *Ensifer*, N225 exhibited the greatest relative abundance of *Opitutus*, the relative abundance of *Mesorhizobium* was peaked in N150 treatment, N75 slightly increased nitrogen-cycling microorganisms relative to N0.

### 3.9 The effects of nitrogen fertilizer inputs on soil biochemical properties, pepino productivity and quality

**FIGURE 9:**
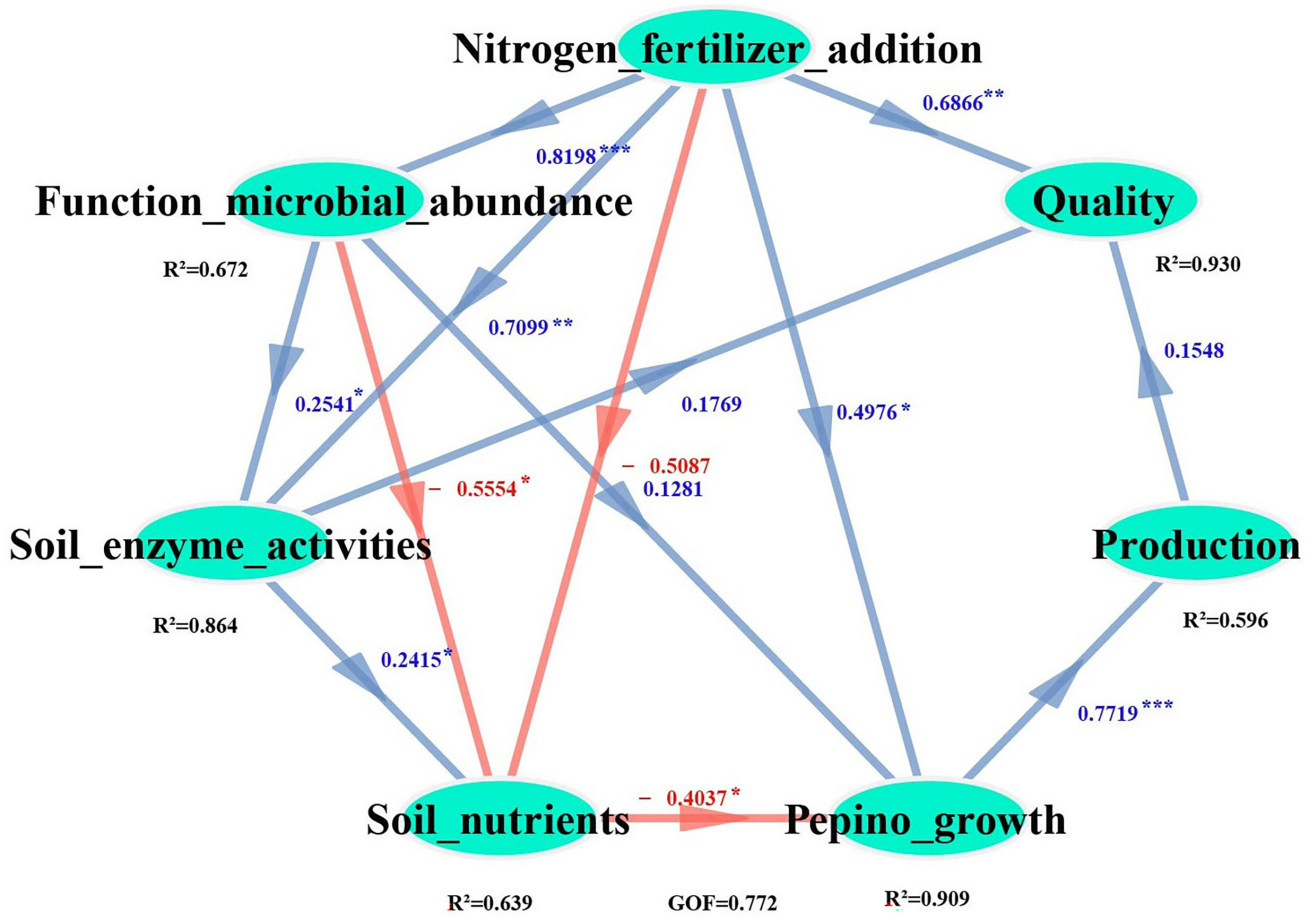
PLS-PM showing the comprehensive effects of nitrogen fertilizer inputs on soil biochemical properties, pepino productivity and quality. The Path coefficients were direct effect. The blue and red arrows represent positive and negative relationships, respectively, and the significance levels are labeled as *(*p* < 0.05), **(*p* < 0.01), ***(*p* < 0.001).

The PLS-PM (Partial Least Squares Path Modeling) analysis revealed that nitrogen fertilizer application enhanced both the relative abundance of dominant bacteria and N-cycling related microorganisms (Supplementary Figure 2; Figure 9). Notably, the relative abundance of functional microorganisms demonstrated a strong correlation with soil urease and nitrate reductase activities. Specifically, nitrogen fertilization indirectly increased pepino production by enhancing soil urease and nitrate reductase activities (PC = 0.71**), as well as increasing the relative abundance of N-cycling related microorganisms (*Nitrosomonas*, *Opitutus*, *Ensifer*, and *Mesorhizobium*) (PC = 0.82***), which facilitated the mobilization of soil nutrients (TN, AN, AK) for pepino growth. In addition, nitrogen fertilization directly improved fruit nutrient parameters (protein and calcium contents) (PC = 0.69**).

## 4 Discussion

### 4.1 Effect of nitrogen fertilizer addition on pepino productivity and fruit nutrient components

Appropriate nitrogen fertilization positively influenced crop yield, whereas excessive application adversely affected crop quality (Park et al., 2023). In our result, both yield per plant and nitrogen use efficiency (NUE) initially increased with rising nitrogen application before stabilizing. Notably, the N225 treatment demonstrated optimal performance in both NUE and yield, compared to N300 treatment (Figures 1b,c), this was consistent with the reasonable application of chemical nitrogen fertilizer enhanced crop nitrogen use efficiency (NUE) and yield (Yang et al., 2020). However, no significant differences in NUE or yield were observed across fertilization levels, possibly due to an insufficiently broad gradient in fertilizer application rates.

Nitrogen fertilization significantly enhanced pepino fruit nutrients, such as protein and calcium (Ca) contents (Table 2), this was consistent with nitrogen deficiency stress (N0) significantly inhibited the biosynthesis of nutrients in L. *barbarum* fruit (Li et al., 2023). Moreover, the N150 treatment yielded the highest soluble sugar content (8.25 g⋅100 g^–1^), while N225 produced the greatest vitamin C concentration (27.65 mg⋅100 g^–1^) (Table 2), this was consistent with excessive nitrogen fertilizer inputs reduced the nutrients contents in L. *barbarum* fruits (Li et al., 2023).

### 4.2 Effect of nitrogen fertilizer application on soil biochemical properties

Soil microbial diversity and composition are primarily governed by pH, organic matter content (SOM), and nutrient availability (Linton et al., 2020; Zhang et al., 2021; Che et al., 2022). Our results demonstrated the N300 treatment induced a 2.01% reduction in soil pH relative to N0, this was consistent with established patterns of nitrogen-mediated soil acidification that negatively impact *Acidobacteriota* and *Chloroflexi* communities (Figure 4a; Rousk and Baath, 2007; Leff et al., 2015). Urea are rapidly converted to ammonium by microbial action in soils, and soil urease activity can be increased, allowing it to hydrolyze urea and increase NH_4_^+^-N content, improving nitrification to form NO_3_^–^-N (Li et al., 2023). In our research, despite the pH was reduced after applying urea to soil, N300 treatment significantly enhanced soil urease and nitrate reductase activities (Figures 2a,b), urease activity stimulation likely linked to the enhancing relative abundance of *Nitrosomonas* (ureolysis) (Figure 8a). *Nitrosomonas*, as ammonia-oxidizing bacteria (AOB), can assimilate the carbon dioxide released by the reaction to make biomass via the Calvin Cycle and harvest energy by oxidizing ammonia (the other product of urease) to nitrate, a process known as nitrification (Marsh et al., 2005). Ammonia oxidizing bacteria (AOB) are critical nodes in the microbial coexistence network and an important participant in the soil nitrogen cycle, catalyzing the first step in the ammonia oxidation process. Meanwhile, the improving of nitrate reductase activity facilitated denitrification to form NO_2_^–^-N, the *Opitutus* (nitrogen reduction) may be participate with this nitrogen cycle process (Figure 8b).

The N225 treatment significantly reduced soil available nitrogen (AN) and potassium (AK) by 29.53 and 27.90%, respectively (Table 3), these reductions may be attributed to enhancing of NUE, yield, and fruit protein, which collectively promoted greater nutrient uptake. Additionally, the observed AK depletion likely contributed to substantial potassium distribution to developing fruits. Notably, nitrogen fertilization increased soil available phosphorus (AP) content (Table 3), this accumulation may result from suppressing of alkaline phosphatase (S_ALP) activity (Figure 2e), and lead to phosphorus (P) accumulation (Sabir et al., 2021).

### 4.3 Effect of chemical nitrogen fertilizer application on soil microbial diversity

Fertilization alters community diversity of soil microorganisms (Sabir et al., 2021). Notably, nitrogen fertilization reduced the diversity of bacterial community (*p* < 0.001) (Li et al., 2019), though some studies reported no significant differences in the α-diversity of rhizosphere bacterial communities under fertilization treatments (Wu et al., 2023). In our study, nitrogen fertilization did not significantly affect the Shannon and Simpson indices (Table 4), but N225 treatment had the greatest bacterial richness (ACE and Chao), the moderate nitrogen application may optimize soil microbial richness (Cruz-Paredes et al., 2021), this was consistent with excessive fertilization reduced α-diversity of bacterial (Li et al., 2023).

### 4.4 Effect of chemical nitrogen fertilizer inputs on soil bacterial community composition

Soil bacterial community exhibits high sensitivity to nutrient inputs, though responses varied significantly among taxa (Francioli et al., 2016). *Proteobacteria* in bacteria participated dominantly and widely in soil nitrogen metabolic processes and had a rich nutritional strategy (Li et al., 2019). Our result demonstrated that nitrogen fertilizer application improved the relative abundance of *Proteobacteria*, *Firmicutes*, and *Bacteroidota*, while decreasing that of *Chloroflexi* and *Acidobacteriota* (Figure 4a), this was consistent with the *Proteobacteria* and *Firmicutes* were copiotrophic taxa with fast growth rates, there were more likely to increase in nutrient-rich conditions. In contrast, *Acidobacteriota* and *Chloroflexi*, classified as oligotrophic and slow-growing, were significantly reduced in fertilizer-treated (Ai et al., 2018; Li et al., 2019; Wu et al., 2023), this likely because they prefer low-nutrient conditions (Fierer et al., 2007; Banerjee et al., 2016; Eo and Park, 2016; Qiu et al., 2021; Wu et al., 2024).

In this research, N150 treatment yielded the highest relative abundance of beneficial genera, including *Bacillus*, *Arthrobacter*, *Sphingomonas*, and *Streptomyces*, these dominance soil-beneficial microbial communities were critical, as they secreted antagonistic compounds that suppress soil-borne pathogens (Salehin et al., 2020; Cheng H. et al., 2021). In addition, *Sphingomonas*, a dominant plant-associated genus, played a vital role in plant-microbe interactions, and was recognized as a plant growth-promoting rhizobacterium (PGPR) (Silva et al., 2018; Cheng C. et al., 2021).

### 4.5 The effects of nitrogen fertilizer inputs on soil biochemical properties, pepino productivity and quality

In our research, the urea inputs significantly impacted on nitrogen cycle-related processes such as ureolysis, nitrogen reduction, nitrogen fixation, this was consistent with previous research (Li et al., 2023). Higher chemical nitrogen fertilizer inputs reduced the relative abundance of *Mesorhizobium*, this due to agroecosystem’s reliance on free-living N-fixing bacteria, stimulate resource competition, and further inhibit nitrogen fixation (Yu et al., 2021).

The PLS-PM analysis revealed that nitrogen fertilizer application indirectly enhanced pepino yield by stimulating urease and nitrate reductase activities, it also promoted the relative abundance of key nitrogen-cycling microorganisms, including *Nitrosomonas* (ureolysis), *Opitutus* (nitrogen reduction), *Ensifer* (nitrogen fixation), *Mesorhizobium* (nitrogen fixation), these microbial communities facilitated the mobilization of critical soil nutrients (TN, AN, AK) to support pepino plant growth. In contrast, fruit nutrients components (such as protein and calcium) were directly modulated by nitrogen fertilizer application rates (Figure 9), this regulation pathway on pepino quality was not consistent with the rate of nitrogen fertilizer (urea) input, the relative abundance of AOB and *Bradyrhizobium*, and their combinations clearly effected the quality (the contents of flavones and vitamins) of L. *barbarum* fruits (Li et al., 2023), which probably due to the different of model types, fruits nutrients components and crop types.

As a result, our research is a vital reference for precise fertilization, nitrogen savings and efficiency improvement in pepino cultivation, and this research lays the foundation for future research into the regulation mechanism of pepino growth on the rhizosphere nitrogen-transforming process through root exudates-mediated.

## 5 Conclusion

To ensure both soil sustainability and economic viability in pepino production, we recommended maintaining nitrogen fertilizer inputs between 150 and 225 kg⋅ha^–1^. Our research demonstrated that nitrogen fertilization indirectly enhanced pepino productivity through enriching functional microorganisms (*Nitrosomonas*, *Ensifer*, *Opitutus*, and *Mesorhizobium*) and stimulating key soil enzyme activities (urease and nitrate reductase) to improve soil nutrient availability, and directly modulated fruit quality through precise nitrogen dosage control. Our study highlighted the importance of optimizing chemical fertilizer management for achieving both production and nutrient objectives in pepino cultivation.

## Data Availability

The original contributions presented in the study are publicly available. This data can be found here: https://www.ncbi.nlm.nih.gov/; accession number PRJNA1300664.

## References

[B1] Ai C. Liang G. Sun J. Wang X. Zhou W. (2012). Responses of extracellular enzyme activities and microbial community in both the rhizosphere and bulk soil to long-term fertilization practices in a fluvo-aquic soil. *Geoderma* 173-174 330–338. 10.1016/j.geoderma.2011.07.020

[B2] Ai C. Zhang S. Zhang X. Guo D. Zhou W. Huang S. (2018). Distinct responses of soil bacterial and fungal communities to changes in fertilization regime and crop rotation. *Geoderma* 319 156–166. 10.1016/j.geoderma.2018.01.010

[B3] Banerjee S. Kirkby C. A. Schmutter D. Bissett A. Kirkegaard J. A. Richardson A. E. (2016). Network analysis reveals functional redundancy and keystone taxa amongst bacterial and fungal communities during organic matter decomposition in an arable soil. *Soil Biol. Biochem.* 97 188–198. 10.1016/j.soilbio.2016.03.017

[B4] Barrios E. (2007). Soil biota, ecosystem services and land productivity. *Ecol. Econ.* 64 269–285. 10.1016/j.ecolecon.2007.03.004

[B5] Berendsen R. L. Pieterse C. M. J. Bakker P. A. H. M. (2012). The rhizosphere microbiome and plant health. *Trends Plant Sci.* 17 478–486. 10.1016/j.tplants.2012.04.001 22564542

[B6] Berg G. Smalla K. (2009). Plant species and soil type cooperatively shape the structure and function of microbial communities in the rhizosphere. *FEMS Microbiol. Ecol*. 68 1–13. 10.1111/j.1574-6941.2009.00654.x 19243436

[B7] Bulgarelli D. Schlaeppi K. Spaepen S. van Themaat E. V. L. Schulze-Lefert P. (2013). Structure and functions of the bacterial microbiota of plants. *Annu. Rev. Plant. Biol.* 64 807–838. 10.1146/annurev-arplant-050312-120106 23373698

[B8] Chao A. (1984). Nonparametric estimation of the number of classes in a population. *Scand. J. Stat.* 11 265–270.

[B9] Che T. Xu Y. Li Y. Wei Z. Zang X. Zhang X. (2022). Mixed planting reduces the shaping ability of legume cover crop on soil microbial community structure. *Appl. Soil Ecol.* 178:104581. 10.1016/j.apsoil.2022.104581

[B10] Chen B. Xiong W. Qi J. Pan H. Chen S. Peng Z. (2021). Trophic interrelationships drive the biogeography of protistan community in agricultural ecosystems. *Soil Biol. Biochem.* 163:104581. 10.1016/j.apsoil.2022.104581

[B11] Chen K. Hu G. Keutgen N. Janssens M. Lenz F. (1999). Effects of NaCl salinity and CO_2_ enrichment on pepino (*Solanum muricatum* Ait.) I. Growth and yield. *Sci. Hortic.* 81 25–41. 10.1016/S0304-4238(98)00264-7

[B12] Chen S. Zhou Y. Chen Y. Gu J. (2018). fastp: An ultra-fast all-in-one FASTQ preprocessor. *Bioinformatics* 34 i884–i890. 10.1093/bioinformatics/bty560 30423086 PMC6129281

[B13] Cheng C. Wang R. Sun L. He L. Sheng X. (2021). Cadmium-resistant and arginine decarboxylase-producing endophytic *Sphingomonas* sp. C40 decreases cadmium accumulation in host rice (Oryza sativa Cliangyou 513). *Chemosphere* 275:130109. 10.1016/j.chemosphere.2021.130109 33677267

[B14] Cheng H. Zhang D. Ren L. Song Z. Li Q. Wu J. (2021). Bio-activation of soil with beneficial microbes after soil fumigation reduces soil-borne pathogens and increases tomato yield. *Environ. Pollut.* 283:117160. 10.1016/j.envpol.2021.117160 33878684

[B15] Contreras C. González-Agüero M. Defilippi B. G. (2016). A review of pepino (*Solanum muricatum* Aiton) fruit: A quality perspective. *HortScience* 51 1127–1133. 10.21273/HORTSCI10883-16

[B16] Contreras C. Schwab W. Mayershofer M. González-Agüero M. Defilippi B. G. (2017). Volatile compound and gene expression analyses reveal temporal and spatial production of LOX-Derived volatiles in pepino (*Solanum muricatum* Aiton) fruit and LOX specificity. *J. Agric. Food Chem.* 65 6049–6057. 10.1021/acs.jafc.7b01569 28669186

[B17] Cruz-Paredes C. Diera T. Davey M. Rieckmann M. M. Christensen P. Dela Cruz M. (2021). Disentangling the abiotic and biotic components of AMF suppressive soils. *Soil Biol. Biochem.* 159:108305. 10.1016/j.soilbio.2021.108305

[B18] Du T. Hu Q. Mao W. Yang Z. Chen H. Sun L. (2023). Metagenomics insights into the functional profiles of soil carbon, nitrogen, and phosphorus cycles in a walnut orchard under various regimes of long-term fertilisation. *Eur. J. Agron.* 148:126887. 10.1016/j.eja.2023.126887

[B19] Edgar R. C. (2013). UPARSE: Highly accurate OTU sequences from microbial amplicon reads. *Nat. Methods* 10 996–998. 10.1038/nmeth.2604 23955772

[B20] Eo J. Park K.-C. (2016). Long-term effects of imbalanced fertilization on the composition and diversity of soil bacterial community. *Agric. Ecosyst. Environ.* 231 176–182. 10.1016/j.agee.2016.06.039

[B21] Fierer N. Bradford M. A. Jackson R. B. (2007). Toward an ecological classification of soil bacteria. *Ecology* 88 1354–1364. 10.1890/05-1839 17601128

[B22] Fierer N. Lauber C. L. Ramirez K. S. Zaneveld J. Bradford M. A. Knight R. (2011). Comparative metagenomic, phylogenetic and physiological analyses of soil microbial communities across nitrogen gradients. *ISME J.* 6 1007–1017. 10.1038/ismej.2011.159 22134642 PMC3329107

[B23] Francioli D. Schulz E. Lentendu G. Wubet T. Buscot F. Reitz T. (2016). Mineral vs. Organic amendments: Microbial community structure, activity and abundance of agriculturally relevant microbes are driven by long-term fertilization strategies. *Front. Microbiol.* 7:1446. 10.3389/fmicb.2016.01446 27683576 PMC5022044

[B24] Franscisco J. Herraiz M. D. R. Santiago Vilanova, María D. García-Martínez, Pietro Gramazio M. P. (2016). Fruit composition diversity in land races and modern pepino (*Solanum muricatum*) varieties and wild related species. *Food Chem.* 203 49–58. 10.1016/j.foodchem.2016.02.035 26948588

[B25] Gao M. Song W. Zhou Q. Ma X. Chen X. (2013). Interactive effect of oxytetracycline and lead on soil enzymatic activity and microbial biomass. *Environ. Toxicol. Pharmacol.* 36 667–674. 10.1016/j.etap.2013.07.003 23892283

[B26] Geisseler D. Scow K. M. (2014). Long-term effects of mineral fertilizers on soil microorganisms – A review. *Soil Biol. Biochem.* 75 54–63. 10.1016/j.soilbio.2014.03.023

[B27] Guo Z. Wan S. Hua K. Yin Y. Chu H. Wang D. (2020). Fertilization regime has a greater effect on soil microbial community structure than crop rotation and growth stage in an agroecosystem. *Appl. Soil Ecol.* 149:103510. 10.1016/j.apsoil.2020.103510

[B28] Heyes J. A. Blaikie F. H. Downs C. G. Sealey D. F. (1994). Textural and physiological changes during pepino (*Solanum muricatum* Ait.) ripening. *Sci. Hortic.* 58 1–15. 10.1016/0304-4238(94)90123-6

[B29] Hou Z. Si C. Zhong Q. Zhang H. Guo Z. Zhang G. (2025). Phenological characteristics and environmental factor associations in open-field cultivation of pepino on the Qinghai-Tibet Plateau. *Int. J. Fruit Sci.* 25 13–27. 10.1080/15538362.2025.2453149

[B30] Hou Z. Sun Z. Du G. Shao D. Zhong Q. Yang S. (2023). Assessment of suitable cultivation region for pepino (*Solanum muricatum*) under different climatic conditions using the MaxEnt model and adaptability in the Qinghai–Tibet plateau. *Heliyon* 9:e18974. 10.1016/j.heliyon.2023.e18974 37636388 PMC10448078

[B31] Hu B. Liang D. Liu J. Lei L. Yu D. (2014). Transformation of heavy metal fractions on soil urease and nitrate reductase activities in copper and selenium co-contaminated soil. *Ecotoxicol. Environ. Saf.* 110 41–48. 10.1016/j.ecoenv.2014.08.007 25193883

[B32] Huyskens-Keil S. Prono-Widayat H. Lüdders P. Schreiner M. (2006). Postharvest quality of pepino (*Solanum muricatum* Ait.) fruit in controlled atmosphere storage. *J. Food Eng.* 77 628–634. 10.1016/j.jfoodeng.2005.07.028

[B33] Ishikawa T. Takahata K. (2019). Insect and mite pests of pepino (*Solanum muricatum* Ait.) in Japan. *Biodivers Data J.* 7:e36453. 10.3897/BDJ.7.e36453 31440114 PMC6702178

[B34] Islam M. R. Singh Chauhan P. Kim Y. Kim M. Sa T. (2010). Community level functional diversity and enzyme activities in paddy soils under different long-term fertilizer management practices. *Biol. Fertility Soils* 47 599–604. 10.1007/s00374-010-0524-2

[B35] Kim O.-K. Ishikawa T. Yamada Y. Sato T. Shinohara H. Takahata K. (2017). Incidence of pests and viral disease on pepino (Solanum muricatum Ait.) in Kanagawa Prefecture, Japan. *Biodivers Data J.* 5:e14879. 10.3897/BDJ.5.e14879 28947875 PMC5592742

[B36] Leff J. W. Jones S. E. Prober S. M. Barberán A. Borer E. T. Firn J. L. (2015). Consistent responses of soil microbial communities to elevated nutrient inputs in grasslands across the globe. *Proc. Natl. Acad. Sci. U S A.* 112 10967–10972. 10.1073/pnas.1508382112 26283343 PMC4568213

[B37] Li Y. Tremblay J. Bainard L. D. Cade-Menun B. Hamel C. (2019). Long-term effects of nitrogen and phosphorus fertilization on soil microbial community structure and function under continuous wheat production. *Environ. Microbiol.* 22 1066–1088. 10.1111/1462-2920.14824 31600863

[B38] Li Y. Zou N. Liang X. Zhou X. Guo S. Wang Y. (2023). Effects of nitrogen input on soil bacterial community structure and soil nitrogen cycling in the rhizosphere soil of *Lycium barbarum* L. *Front. Microbiol.* 13:1070817. 10.3389/fmicb.2022.1070817 36704567 PMC9871820

[B39] Linton N. F. Ferrari Machado P. V. Deen B. Wagner-Riddle C. Dunfield K. E. (2020). Long-term diverse rotation alters nitrogen cycling bacterial groups and nitrous oxide emissions after nitrogen fertilization. *Soil Biol. Biochem.* 149:107917. 10.1016/j.soilbio.2020.107917

[B40] Liu C. Zhao D. Ma W. Guo Y. Wang A. Wang Q. (2015). Denitrifying sulfide removal process on high-salinity wastewaters in the presence of *Halomonas* sp. *Appl. Microbiol. Biotechnol.* 100 1421–1426. 10.1007/s00253-015-7039-6 26454867

[B41] Ma R. Cao N. Li Y. Hou Y. Wang Y. Zhang Q. (2024). Rational reduction of planting density and enhancement of NUE were effective methods to mitigate maize yield loss due to excessive rainfall. *Eur. J. Agron.* 160:127326. 10.1016/j.eja.2024.127326

[B42] Magoč T. Salzberg S. L. (2011). FLASH: Fast length adjustment of short reads to improve genome assemblies. *Bioinformatics* 27 2957–2963. 10.1093/bioinformatics/btr507 21903629 PMC3198573

[B43] Manuel Delgado-Baquerizo M. Maestre F. T. Reich P. B. Trivedi P. Osani Y. Liu Y. (2016). Carbon content and climate variability drive global soil bacterial diversity patterns. *Ecol. Monogr.* 86 373–390. 10.1002/ecm.1216

[B44] Marsh K. L. Sims G. K. Mulvaney R. L. (2005). Availability of urea to autotrophic ammonia-oxidizing bacteria as related to the fate of ^14^C- and ^15^N-labeled urea added to soil. *Biol. Fertility Soils* 42 137–145. 10.1007/s00374-005-0004-2

[B45] Martínez-Romero D. (2003). Physiological changes in pepino (*Solanum muricatum* Ait.) fruit stored at chilling and non-chilling temperatures. *Postharvest Biol. Technol.* 30 177–186. 10.1016/S0925-5214(03)00106-6

[B46] Ouyang Y. Norton J. M. Parales R. E. (2020). Short-term nitrogen fertilization affects microbial community composition and nitrogen mineralization functions in an agricultural soil. *Appl. Environ. Microbiol.* 86:e02278-19. 10.1128/AEM.02278-19 31836579 PMC7028969

[B47] Pacheco J. Plazas M. Pettinari I. Landa-Faz A. González-Orenga S. Boscaiu M. (2021). Moderate and severe water stress effects on morphological and biochemical traits in a set of pepino (*Solanum muricatu*m) cultivars. *Sci. Hortic.* 284:110143. 10.1016/j.scienta.2021.110143

[B48] Park J.-R. Jang Y.-H. Kim E.-G. Lee G.-S. Kim K.-M. (2023). Nitrogen fertilization causes changes in agricultural characteristics and gas emissions in rice field. *Sustainability* 15:3336. 10.3390/su15043336

[B49] Prohens J. Rodríguez-Burruezo A. Nuez F. (2005). Utilization of genetic resources for the introduction and adaptation of exotic vegetable crops: The case of pepino (*Solanum muricatum*). *Euphytica* 146 133–142. 10.1007/s10681-005-3882-3

[B50] Qiu L. Zhang Q. Zhu H. Reich P. B. Banerjee S. van der Heijden M. G. A. (2021). Erosion reduces soil microbial diversity, network complexity and multifunctionality. *ISME J.* 15 2474–2489. 10.1038/s41396-021-00913-1 33712698 PMC8319411

[B51] Ramirez K. S. Craine J. M. Fierer N. (2012). Consistent effects of nitrogen amendments on soil microbial communities and processes across biomes. *Glob. Change Biol.* 18 1918–1927. 10.1111/j.1365-2486.2012.02639.x

[B52] Ren T. Feng H. Xu C. Xu Q. Fu B. Azwar E. (2022). Exogenous application and interaction of biochar with environmental factors for improving functional diversity of rhizosphere’s microbial community and health. *Chemosphere* 294:133710. 10.1016/j.chemosphere.2022.133710 35074326

[B53] Rousk J. Baath E. (2007). Fungal and bacterial growth in soil with plant materials of different C/N ratios. *FEMS Microbiol. Ecol.* 62 258–267. 10.1111/j.1574-6941.2007.00398.x 17991019

[B54] Sabir M. S. Shahzadi F. Ali F. Shakeela Q. Niaz Z. Ahmed S. (2021). Comparative effect of fertilization practices on soil microbial diversity and activity: An overview. *Curr. Microbiol.* 78 3644–3655. 10.1007/s00284-021-02634-2 34480627

[B55] Salehin A. Hafiz M. Hayashi S. Adachi F. Itoh K. (2020). Effects of the biofertilizer OYK (*Bacillus* sp.) inoculation on endophytic microbial community in sweet potato. *Horticulturae* 6:81. 10.3390/horticulturae6040081

[B56] Schaffer I. R. Rylski I. Fogelman M. (1989). Carbohydrate content and sucrose metabolism in developing *Solanum muricatum* fruits. *Phytochemistry* 28 737–739. 10.1016/0031-9422(89)80105-0

[B57] Schloss P. D. Westcott S. L. Ryabin T. Hall J. R. Hartmann M. Hollister E. B. (2009). Introducing mothur: Open-source, platform-independent, community-supported software for describing and comparing microbial communities. *Appl. Environ. Microbiol.* 75 7537–7541. 10.1128/AEM.01541-09 19801464 PMC2786419

[B58] Shannon C. E. (1948). A mathematical theory of communication. *Bell Syst. Techn. J.* 27 379–423. 10.1002/j.1538-7305.1948.tb01338.x

[B59] She W. Bai Y. Zhang Y. Qin S. Feng W. Sun Y. (2018). Resource availability drives responses of soil microbial communities to short-term precipitation and nitrogen addition in a desert shrubland. *Front. Microbiol.* 9:186. 10.3389/fmicb.2018.00186 29479346 PMC5811472

[B60] She X. Yu L. Lan G. Tang Y. Deng M. Li Z. (2021). Pantoea agglomerans causing blight disease on pepino melon (*Solanum muricatum*) in China. *Crop Protection* 139:105385. 10.1016/j.cropro.2020.105385

[B61] Silva J. C. P. D. Medeiros F. H. V. D. Campos V. P. (2018). Building soil suppressiveness against plant-parasitic nematodes. *Biocontrol Sci. Technol.* 28 423–445. 10.1080/09583157.2018.1460316

[B62] Simpson E. H. (1949). Measurement of diversity. *Nature* 163:688. 10.1038/163688a0

[B63] Stackebrandt E. Goebel B. M. (1994). Taxonomic note: A place for DNA-DNA reassociation and 16S rRNA sequence analysis in the present species definition in bacteriology. *Int. J. Syst. Bacteriol.* 44 846–849. 10.1099/00207713-44-4-846

[B64] Sudha G. Sangeetha Priya M. Indhu Shree R. B. Vadivukkarasi S. (2012). Antioxidant activity of ripe and unripe pepino fruit (*Solanum muricatum* Aiton). *J. Food Sci.* 77 C1131–C1135. 10.1111/j.1750-3841.2012.02944.x 23057510

[B65] Sun Z. Wang L. Zhang G. Yang S. Zhong Q. (2022). Pepino (*Solanum muricatum*) metabolic profiles and soil nutrient association analysis in three growing sites on the Loess Plateau of Northwestern China. *Metabolites* 12:885. 10.3390/metabo12100885 36295787 PMC9610035

[B66] Vries W. D. Liu X. Yuan L. (2022). Highlights of the special issue “progress on nitrogen research: From plant, soil to the environment”. *Front. Agric. Sci. Eng.* 9:313. 10.15302/J-FASE-2022460

[B67] Walkley A. (1935). An examination of methods for determining organic carbon and nitrogen in soils. *J. Agric. Sci.* 25 598–609. 10.1017/S0021859600019687

[B68] Wang J. Rhodes G. Huang Q. Shen Q. (2018). Plant growth stages and fertilization regimes drive soil fungal community compositions in a wheat-rice rotation system. *Biol. Fertility Soils* 54 731–742. 10.1007/s00374-018-1295-4

[B69] Wang Q. Garrity G. M. Tiedje J. M. Cole J. R. (2007). Naïve Bayesian Classifier for rapid assignment of rRNA sequences into the new bacterial taxonomy. *Appl. Environ. Microbiol.* 73 5261–5267. 10.1128/AEM.00062-07 17586664 PMC1950982

[B70] Wu C. Yan B. Wei F. Wang H. Gao L. Ma H. (2023). Long-term application of nitrogen and phosphorus fertilizers changes the process of community construction by affecting keystone species of crop rhizosphere microorganisms. *Sci. Total Environ.* 897:165239. 10.1016/j.scitotenv.2023.165239 37394065

[B71] Wu Q. Chen Y. Dou X. Liao D. Li K. An C. (2024). Microbial fertilizers improve soil quality and crop yield in coastal saline soils by regulating soil bacterial and fungal community structure. *Sci. Total Environ.* 949:175127. 10.1016/j.scitotenv.2024.175127 39084360

[B72] Xu W. Prieme A. Cooper E. J. Mörsdorf M. A. Semenchuk P. Elberling B. (2021). Deepened snow enhances gross nitrogen cycling among Pan-Arctic tundra soils during both winter and summer. *Soil Biol. Biochem.* 160:108356. 10.1016/j.soilbio.2021.108356

[B73] Yadav S. K. Soni R. Rajput A. S. (2018). *Role of microbes in organic farming for sustainable agro-ecosystem, Microorganisms for green revolution.* Singapore: Springer Nature Singapore Pte Ltd, 241–252.

[B74] Yang S. Sun Z. Zhang G. Wang L. Zhong Q. (2023). Identification of the key metabolites and related genes network modules highly associated with the nutrients and taste components among different pepino (*Solanum muricatum*) cultivars. *Food Res. Int.* 163:112287. 10.1016/j.foodres.2022.112287 36596193

[B75] Yang W. Gong T. Wang J. Li G. Liu Y. Zhen J. (2020). Effects of compound microbial fertilizer on soil characteristics and yield of wheat (*Triticum aestivum* L.). *J. Soil Sci. Plant Nutr.* 20 2740–2748. 10.1007/s42729-020-00340-9

[B76] Yang Y. Xie H. Mao Z. Bao X. He H. Zhang X. (2022). Fungi determine increased soil organic carbon more than bacteria through their necromass inputs in conservation tillage croplands. *Soil Biol. Biochem.* 167:108587. 10.1016/j.soilbio.2022.108587

[B77] Yu L. Luo S. Gou Y. Xu X. Wang J. (2021). Structure of rhizospheric microbial community and N cycling functional gene shifts with reduced N input in sugarcane-soybean intercropping in South China. *Agric. Ecosyst. Environ.* 314:107413. 10.1016/j.agee.2021.107413

[B78] Yue H. Xu Q. Bian G. Guo Q. Fang Z. Wu W. (2020). Structure characterization and immunomodulatory activity of a new neutral polysaccharide SMP-0b from *Solanum muricatum*. *Int. J. Biol. Macromol.* 155 853–860. 10.1016/j.ijbiomac.2019.11.071 31712159

[B79] Zeng J. Liu X. Song L. Lin X. Zhang H. Shen C. (2016). Nitrogen fertilization directly affects soil bacterial diversity and indirectly affects bacterial community composition. *Soil Biol. Biochem.* 92 41–49. 10.1016/j.soilbio.2015.09.018

[B80] Zhang Y. Xu X. Li Z. Xu C. Luo W. (2021). Improvements in soil quality with vegetation succession in subtropical China karst. *Sci. Total Environ.* 775:145876. 10.1016/j.scitotenv.2021.145876 33631590

[B81] Zhao Y. Zuo J. Yuan S. Shi W. Shi J. Feng B. (2021). UV-C treatment maintains the sensory quality, antioxidant activity and flavor of pepino fruit during postharvest storage. *Foods* 10:2964. 10.3390/foods10122964 34945515 PMC8701303

[B82] Zhou G. Fan K. Gao S. Chang D. Li G. Liang T. (2023). Green manuring relocates microbiomes in driving the soil functionality of nitrogen cycling to obtain preferable grain yields in thirty years. *Sci. China Life Sci.* 67 596–610. 10.1007/s11427-023-2432-9 38057623

