# Supplementary figures and images for: Indirect improvement of pepino (Solanum muricatum) productivity via nitrogen fertilizer-mediated microbial and enzymatic stimulation
Source: Front Microbiol. 2025 Aug 14;16:1612012. doi: 10.3389/fmicb.2025.1612012 (PMC12391131; doi:10.3389/fmicb.2025.1612012)

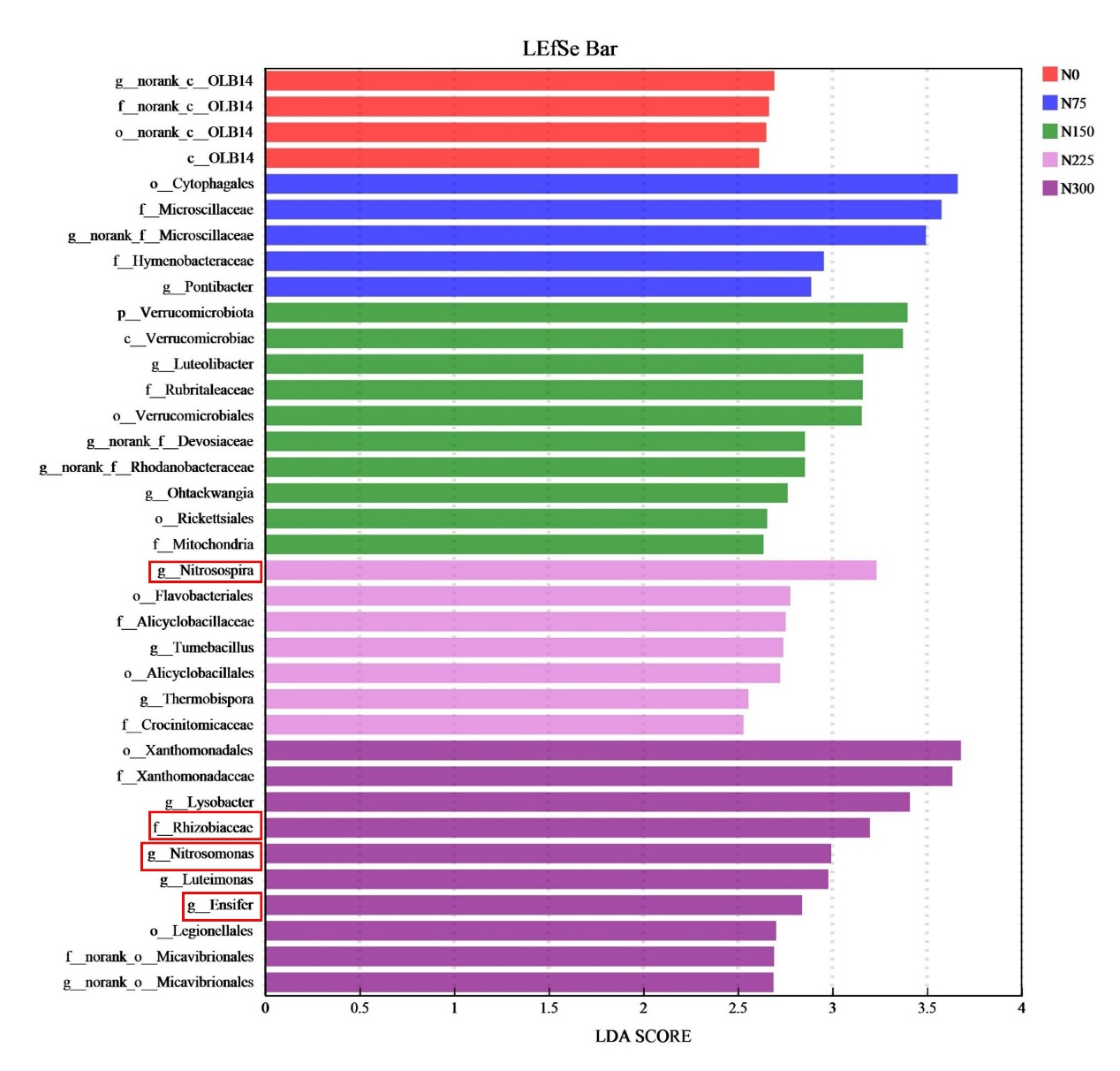


**Fig.1_supplement**


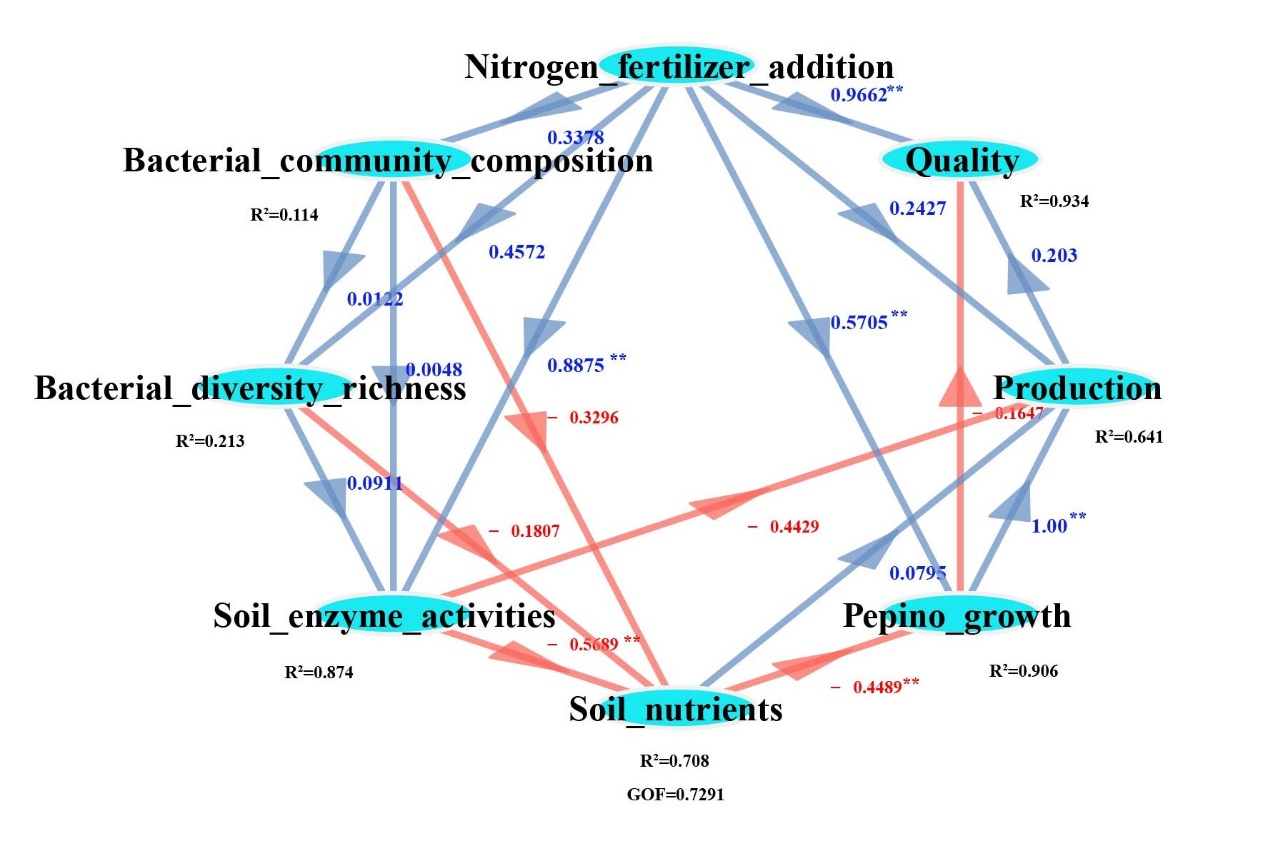


**Fig.2_supplement**

Supplement: Supplementary file 1 [file Table_1.docx]
