# Supplementary material for: Indirect improvement of pepino (Solanum muricatum) productivity via nitrogen fertilizer-mediated microbial and enzymatic stimulation
Source: Front Microbiol. 2025 Aug 14;16:1612012. doi: 10.3389/fmicb.2025.1612012 (PMC12391131; doi:10.3389/fmicb.2025.1612012)
Supplement: Supplementary file 2 [file Table_2.docx]

**Table 1_supplement is the conversion of measurement units for nitrogen fertilizer amounts**

| **Measurement Units** | **N0** | **N75** | **N150** | **N225** | **N300** |
| --- | --- | --- | --- | --- | --- |
| **kg·ha⁻¹** | **0 kg·ha⁻¹** | **75 kg·ha⁻¹** | **150 kg·ha⁻¹** | **225 kg·ha⁻¹** | **300 kg·ha⁻¹** |
| **kg·mu^-1^** | **0 kg·mu^-1^** | **5 kg·mu^-1^** | **10 kg·mu^-1^** | **15 kg·mu^-1^** | **20 kg·mu^-1^** |
| **g· (kg soil)^-1^** | **0 g· (kg soil)^-1^** | **0.033 g****· (kg soil)^-1^** | **0.067 g· (kg soil)^-1^** | **0.100 g· (kg soil)^-1^** | **0.133 g· (kg soil)^-1^** |
| **g· (10 kg soil)^-1^** | **0** **g· (10 kg soil)^-1^** | **0.33 g· (10 kg soil)^-1^** | **0.67 g· (10 kg soil)^-1^** | **1.00 g· (10 kg soil)^-1^** | **1.33 g· (10 kg soil)^-1^** |
